# In vitro and in silico studies of enterobactin-inspired Ciprofloxacin and Fosfomycin first generation conjugates on the antibiotic resistant *E. coli* OQ866153

**DOI:** 10.1186/s12866-024-03248-x

**Published:** 2024-03-22

**Authors:** Mohamed T. Khazaal, Ahmed H. I. Faraag, Hoda H. El-Hendawy

**Affiliations:** 1 Botany and Microbiology Department, Faculty of Science, Helwan University, HelwanCairo, 11795 Egypt; 2 School of Biotechnology, Badr University in Cairo, Badr City, Cairo, 11829 Egypt

**Keywords:** Antimicrobial therapy, Enterobactin, Siderophores, Siderophore-drug conjugate "Trojan Horse" strategy

## Abstract

**Background:**

The emergence of antimicrobial resistance in bacterial pathogens is a growing concern worldwide due to its impact on the treatment of bacterial infections. The "Trojan Horse" strategy has been proposed as a potential solution to overcome drug resistance caused by permeability issues.

**Objective:**

The objective of our research was to investigate the bactericidal activity and mechanism of action of the "Trojan Horse" strategy using enterobactin conjugated with Ciprofloxacin and Fosfomycin against the antibiotic-resistant Escherichia coli strain OQ866153.

**Methodology:**

Enterobactin, a mixed ligand of *E. coli* OQ866153, was conjugated with Ciprofloxacin and Fosfomycin individually to aid active absorption via specific enterobactin binding proteins (*FepABCDG*). The effectiveness of the conjugates was assessed by measuring their bactericidal activity against *E. coli* OQ866153, as well as their ability to inhibit DNA *gyrase* enzyme and biofilm formation.

**Results:**

The Fe^+3^-enterobactin-Ciprofloxacin conjugate effectively inhibited the DNA *gyr*ase enzyme (Docking score = -8.597 kcal/mol) and resulted in a lower concentration (25 μg/ml) required to eliminate supercoiled DNA plasmids compared to the parent drug (35 μg/ml; Docking score = -6.264 kcal/mol). The Fe^+3^-Enterobactin-Fosfomycin conjugate showed a higher inhibition percentage (100%) of biofilm formation compared to Fosfomycin (21.58%) at a concentration of 2 mg/ml, with docking scores of -5.481 and -3.756 kcal/mol against UDP-N acetylglucosamine 1-carboxyvinyltransferase *MurA*.

**Conclusion:**

The findings of this study suggest that the "Trojan Horse" strategy using enterobactin conjugated with Ciprofloxacin and Fosfomycin can effectively overcome permeability issues caused by efflux proteins and enhance the bactericidal activity of these drugs against antibiotic-resistant strains of *E. coli*.

**Supplementary Information:**

The online version contains supplementary material available at 10.1186/s12866-024-03248-x.

## Background

Antibiotic resistance has become a global health crisis, with the rise of drug-resistant bacteria posing a significant threat to public health. Anticipated outcomes of antimicrobial resistance are immense, with an estimated 50 million deaths and a $100 trillion cost to the global economy projected by 2050 [1]. In particular, approximately 2 million deaths occur annually due to pathogenic *Escherichia coli* (*E. coli*), which cause a variety of human and foodborne diseases, especially in developing countries [2]. The overuse and incorrect usage of antibiotics for the treatment of bacterial infections have led to a significant rise in bacterial resistance to nearly all available antibiotics. Moreover, efflux pumps overexpressed by many Gram-negative bacteria play an eminent role in their protection against antimicrobial drugs [3].

Various national and international agencies have made concerted efforts to manage and combat drug resistance. These agencies recognize the urgent need for coordinated actions to preserve the efficacy of existing antibiotics and promote the development of new therapeutic interventions [4, 5]. One of the key global initiatives is the World Health Organization's Global Action Plan on Antimicrobial Resistance (AMR). This comprehensive plan, adopted in 2015, aims to combat drug resistance through a multifaceted approach that includes improving awareness and understanding, strengthening surveillance and research, reducing the incidence of infection, optimizing the use of antimicrobial agents, and increasing investment in new drug development [6]. Additionally, regional organizations such as the European Centre for Disease Prevention and Control (ECDC) and the Centers for Disease Control and Prevention (CDC) in the United States have been actively involved in addressing antibiotic resistance. These agencies provide guidelines, conduct surveillance, and support research to monitor and control the spread of drug-resistant bacteria [7]. Furthermore, national governments have implemented various strategies to combat antibiotic resistance. For example, in the United Kingdom, the National Health Service (NHS) launched the "Keep Antibiotics Working" campaign to raise public awareness about the appropriate use of antibiotics and the consequences of misuse [7–10]. Similarly, countries like Sweden and the Netherlands have implemented strict regulations on antibiotic use in both humans and animals, resulting in significant reductions in antibiotic consumption and resistance rates [11, 12].

In recent years, due to the limited number of new antibiotics being discovered, there has been a need to develop creative methods (novel antibiotics, delivery systems, and bacterial diagnostics) to overcome effective efflux barriers (such as, *AcrB* and *TolC*) and fight against harmful bacteria. One such approach that has shown promise is the bacterial siderophore Trojan horse strategy, where antibiotics are bound to siderophores. In this way, as the bacterial cell transports the iron-bound siderophore, which is vital for its survival, it inadvertently takes up the antibiotic into the periplasm [13]**.** The effectiveness of antibiotics is enhanced by circumventing the outer-membrane barrier [14]**.**

The initial step in microbial iron uptake involves the cell producing and releasing "endogenous" siderophores, which are compounds (specifically catecholate, hydroxamate, and α-hydroxycarboxylate ligands) that the cell itself creates. These siderophores are then released into the surrounding environment to bind to iron (Fe), either by solubilizing Fe (III) from its hydroxide form or by capturing it from host proteins [15]**.**

Gram-negative bacteria typically rely on specialized transport mechanisms in their outer membrane to facilitate the entry of siderophore-Fe (III) complexes into cells. This is necessary because these complexes tend to have a molecular weight that exceeds 500 Da [16].

Upon recognition, the transport system becomes activated and facilitates the uptake of the substance. *E. coli* has been found to possess at least nine iron transport systems, including the *fhu* system for ferrichrome, the *fep* system for enterobactin (also known as enterochelin, a cyclic trimer of 2,3-dihydroxybenzoyl-L-serine produced by bacteria such as *E. coli* belonging to the family Enterobacteriaceae), the *fec* system for ferric citrate, the *iut* system for aerobactin, the *chu* system for heme, the *fhuE* system for rhodotorulic acid and coprogen, the *iro* system for salmochelin, the yersiniabactin system, and the *feo* system for ferrous iron [17]**.**

The outer membrane receptors of ferric siderophores provide nourishment to cells, but they can also have harmful effects. Some microorganisms have developed a strategy using siderophores and a bactericidal component called sideromycin to specifically target and eliminate other species. This approach, similar to the Trojan horse tactic, has motivated scientists to develop a new method for enhancing drug penetration into cells that are resistant to multiple drugs [18]**.** In this case, the bactericidal activity of a synthetic muramycin, which inhibits the essential *MraY* protein in bacterial membrane peptidoglycan biosynthesis, was greatly enhanced when conjugated with enterobactin. This improvement was particularly notable against an *E. coli* strain with a deficient *TolC* efflux pump, as these conjugates typically face challenges in cellular uptake in Gram-negative bacteria [3]**.**

In this study, the synthesis and efficacy of enterobactin-Ciprofloxacin and Fosfomycin conjugates were presented, which were designed to act as Trojan Horse antimicrobials. These conjugates were specifically developed to target antibiotic-resistant *E. coli* strains by utilizing the enterobactin transport machinery. By binding to siderophore-bind proteins *FepABCDG*, they were able to overcome the permeability issues caused by efflux proteins *AcrB* and *TolC*. The conjugates demonstrated enhanced effectiveness against *E. coli* OQ866153 by inhibiting DNA gyrase and replication and also by inhibiting biofilm formation through UDP-N-acetylglucosamine1-carboxyvinyltransferase (*MurA*).

## Results

### Detection of enterobactin-binding, cell membrane efflux proteins, DNA *gyr*ase, and *MurA* genes

**Fig. 1:**
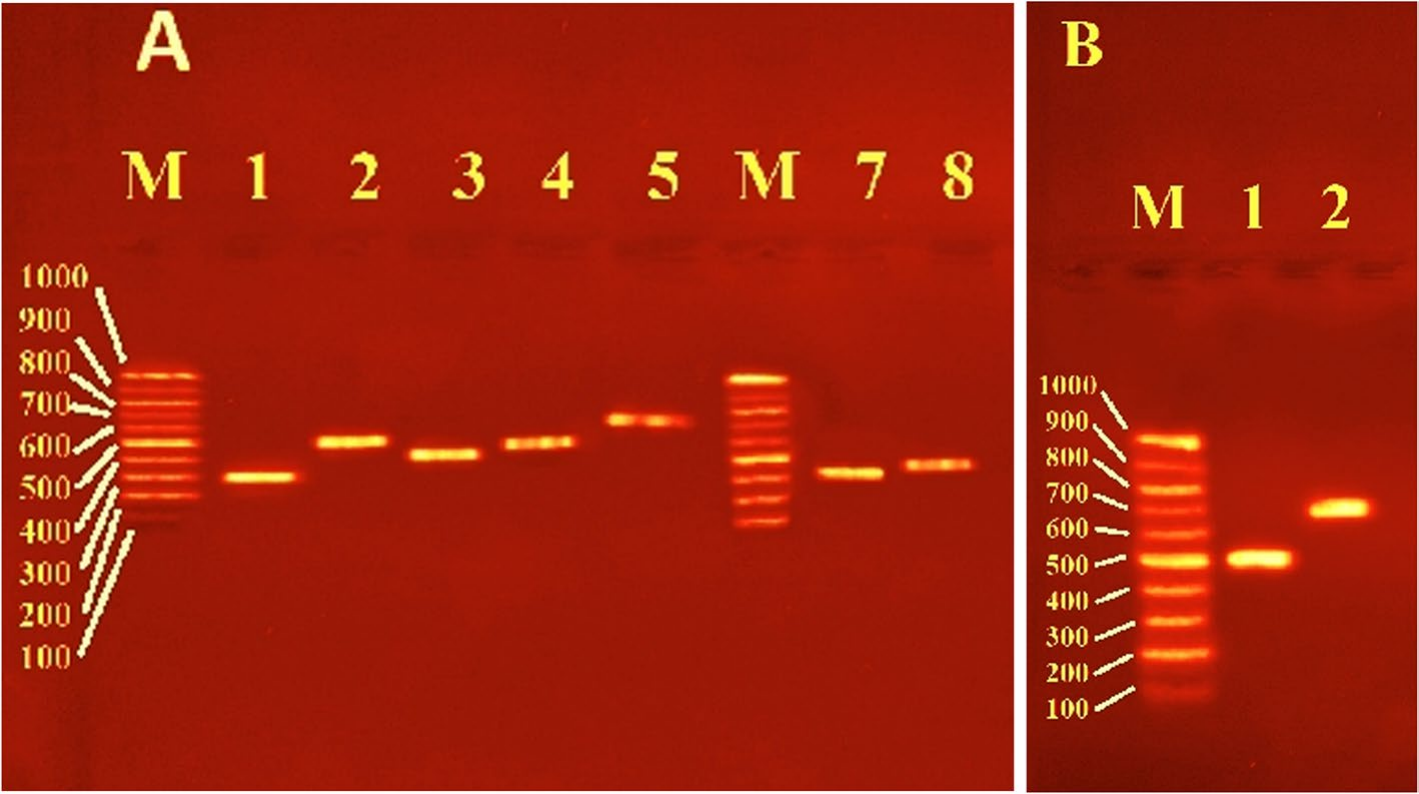
Agarose gel electrophoresis of *E. coli* OQ866153 strain. **A**, enterobactin-binding and efflux proteins genes amplified by PCR. Lane M: 100 bp molecular weight DNA marker. lanes 1, *fepA;* 2, *fepB;* 3, *fepC;* 4, *fepD*; 5, *fepG;* 7, *AcrB*; and 8, *TolC*, respectively. **B**, DNA *gyr*ase and *MurA* PCR amplicons. Lanes 1, DNA *gyr*ase and 2, *MurA*

PCR amplification of enterobactin-binding, cell membrane efflux proteins, DNA *gyr*ase, and *MurA* genes was carried out using two oligonucleotide primers for each gene (Table 1) and the amplicons were examined using agarose gel electrophoresis (Fig. 1A, B). The genes for the enterobactin-binding proteins *FepA*, *FepB*, *FepC*, *FepD*, and *FepG* had lengths of 366, 503, 451, 481, and 649 bp, respectively. The purified PCR products of the efflux protein genes *AcrB* and *TolC* were 358 and 388 bp, respectively. Additionally, the purified PCR products of the DNA gyrase and *MurA* genes were 500 and 710 bp in size, respectively.

**Table 1:** Designed primers and PCR products size of enterobactin binding, efflux proteins, DNA *gyr*ase, and *MurA* genes

Gene		product	PCR product size (bp)	Primer		Ta (°C)
**Enterochelin binding protein**	***FepA***	siderophore enterobactin receptor *FepA*	366	714F21	5' GTATGCCACGACGTTACCAGC 3'	59.0
				1060R20	5' ACGAAATCCTGTGTCGCTTT 3'	
	***FepB***	Fe^2+^-enterobactin ABC transporter substrate binding protein	503	291F21	5' ACTGCAACGGCTCTATATCGG 3'	59.2
				773R21	5' ACAGGAATAGTGACTCGCCAT 3'	
	***FepC***	iron-enterobactin ABC transporter ATP-binding protein	451	313F21	5' GGACGTTATCCGCATCAACCG 3'	59.0
				744R20	5' TTCCGGCAACTGGATCGTCA 3'	
	***FepD***	Fe^3+^ -siderophore ABC transporter permease	481	434F25	5' AGTTAAGTCCGGTGCGTTTAACCCT 3'	62.1
				894R21	5’ GGCACAATCACCCGCCCGATG 3’	
	***FepG***	iron-enterobactin ABC transporter permease	649	12F23	5’ CTCTCGCCGATTACTCATCACCT 3’	61.5
				639R22	5' GCCCATTTCCAGCAAGCGCATC 3'	
***Efflux proteins***	***AcrB***	efflux RND transporter permease *AcrB*	358	680F20	5' GCCAACAGCTTAACGCCTCT 3'	58.6
				1017R21	5' TCGACCAGCGTTTTAACCACT 3'	
	***TolC***	outer membrane channel protein *TolC*	388	97F20	5' CAAGCACGCCTTAGTAACCC 3'	56.5
				463R22	5' CTAATTGACGGTAGATCGCTTC 3'	
**DNA *gyr*ase**		DNA topoisomerase (ATP-hydrolyzing) subunit B	500	183F23	5' CACCATTCACGCCGATAACTCTG 3'	59.9
				661R22	5' ATTCAACGAACGCCTTGATGCC 3'	
***MurA***		UDP-N acetylglucosamine 1-carboxyvinyltransferase	710	393F24	5' ATTAGGCGCGACCATCAAACTGGA 3'	60.7
				1081R22	5' TTGCCATAACCTGTGCGCCAGA 3'	

bp, base pair; Ta, annealing temperature

### Trojan-Horse strategy

#### Preparation of enterobactin-antibiotic conjugates

The primary absorbance bands in the FTIR spectrum of pure Ciprofloxacin were observed at 3529, 3375, 3085, 2918, 2687, 2619, 1702, 1622, 1492, 1447, 1383, 1341, and 1269 cm^−1^ (Fig. S2A). The most significant band, corresponding to the carbonyl group, was found at 1702 cm^−1^. The IR spectrum bands (941, 1049, 1115, 1279, 1428, 1461, 2941, 3326, and 3560 cm^−1^) obtained were nearly identical to those of commercially available Fosfomycin sodium salt (Fig. S2B). The characteristic peaks around 1049 and 1115 cm − 1 indicated stretching vibrations of (PO_3_)^2−^ and (C–O–C), respectively. The IR spectrum of the Fe^+3^-enterobactin complex resembled that of enterobactin.

**Fig. 2:**
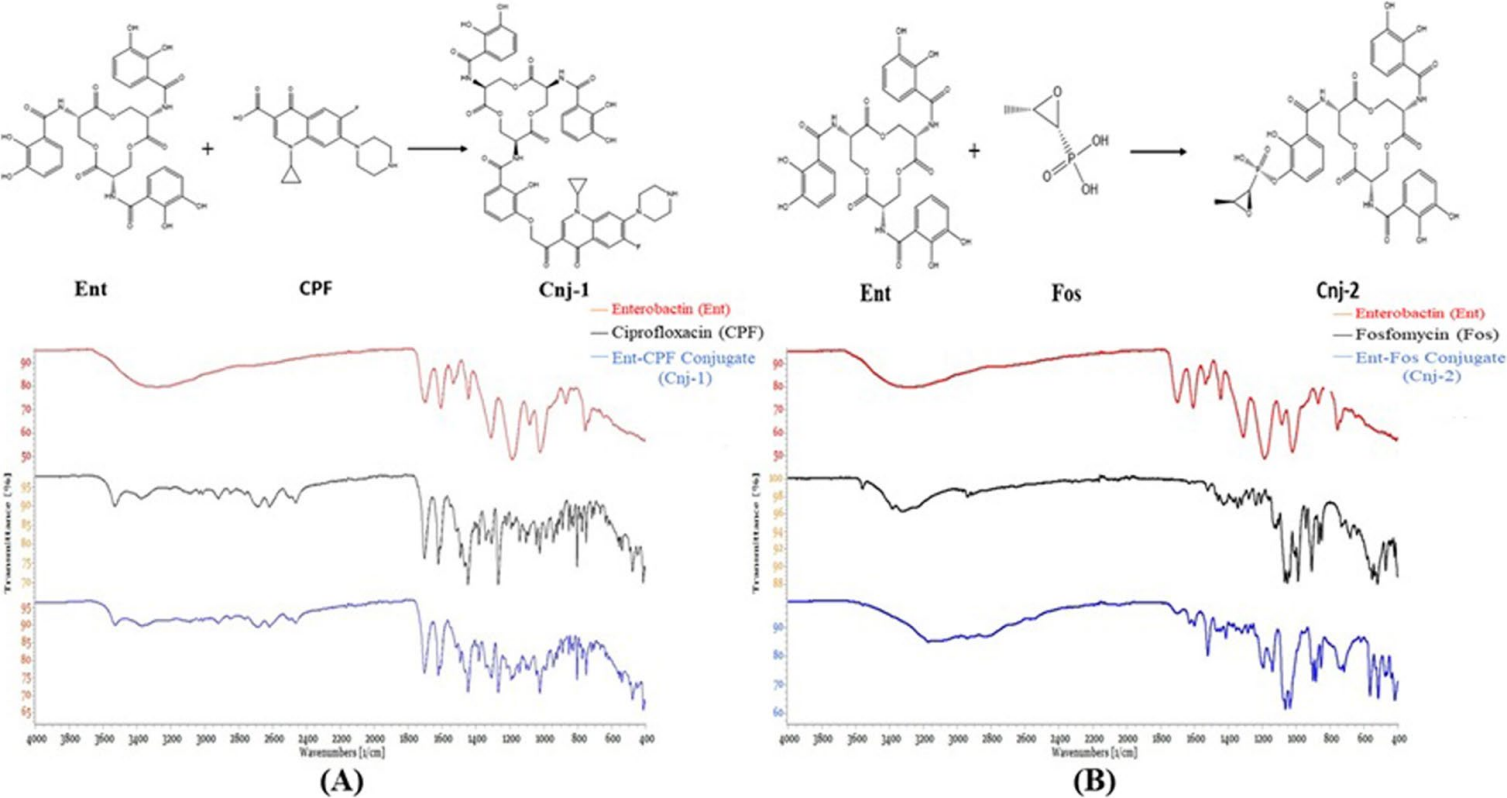
FTIR spectrum at a 400–4000 cm-1 wavelength range with a resolution of 4 cm^−1^ showing interaction between enterobactin (Ent) and, (**A**), Ciprofloxacin (CIP) forming Ent-CPF conjugate (Cnj-1) and (**B**), Fosfomycin (Fos) forming Ent-Fos conjugate (Cnj-2), at (-NH) and (-OH) positions of enterobactin, respectively

Figure 2A shows the presence of the antisymmetric (-NH) stretching band at 3000–3500 cm^−1^ in enterobactin, both with and without Ciprofloxacin. When Ciprofloxacin was loaded into enterobactin, the disappearance of this band indicated an interaction between the two at the (-NH) position. Figure 2B shows that the antisymmetric stretching bands (-OH) at 1185 and 1500 cm^−1^ also disappeared in the enterobactin sample, suggesting interaction between enterobactin and Fosfomycin at the (-OH) position. Additionally, complexes prepared at pH 9 were found to be more stable compared to those prepared at pH 4 and 7.

### In Vitro antibacterial studies

#### Determination of minimum inhibitory concentrations (MICs) by broth microdilution assay

To assess the effectiveness of Fe^+3^-enterobactin-Ciprofloxacin/Fosfomycin conjugates in inhibiting the growth of *E. coli* OQ866153, The MIC of each conjugate was examined at various concentrations (1000, 500, 250, 125, 62, 31.25, 7.8, 3.9, 1.9, and 0.97 µg/ml) after a 24-h incubation period. The MIC values for Fe^+3^-Cnj-1 and Fe^+3^-Cnj-2 conjugates were 31.25 and 500 µg/ml, respectively. These results indicate that the conjugates have strong activity against *E. coli* OQ866153. Hence, a study was conducted to investigate the antibacterial mechanism employed by these conjugates against a resistant strain of *E. coli* (OQ866153).

#### Time kill assay

**Fig. 3:**
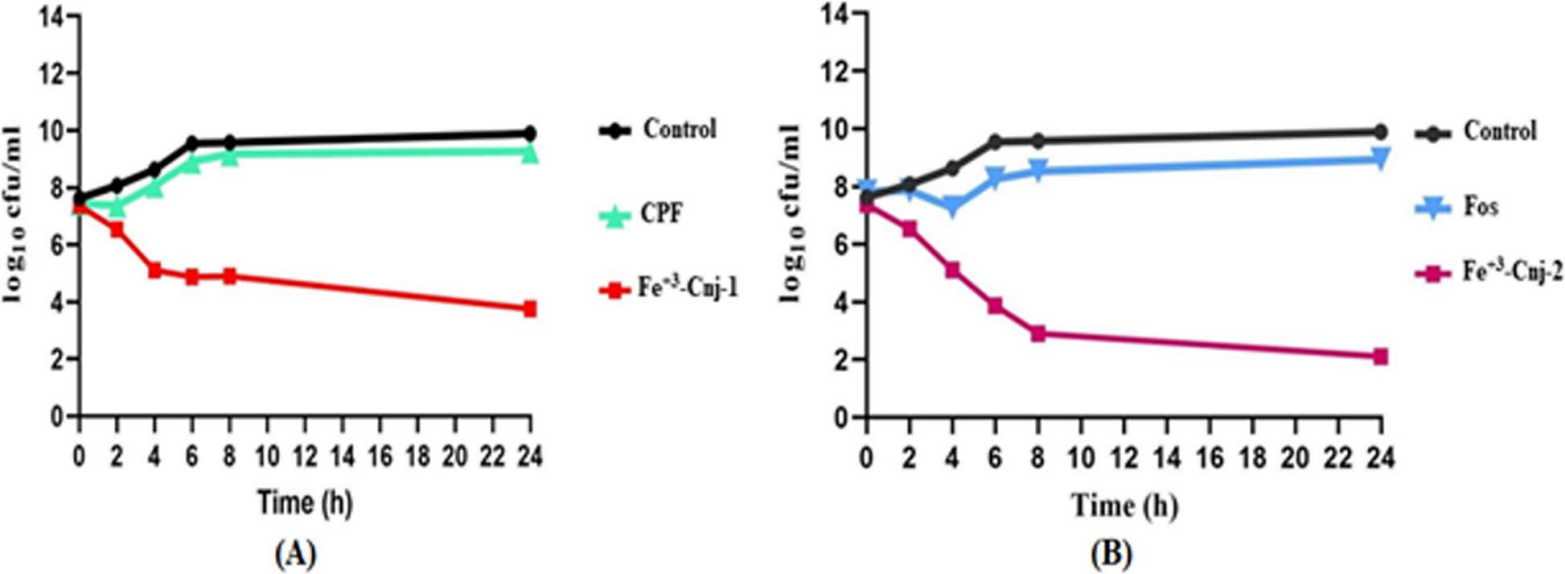
Time kill kinetics of (**A**), Fe^+3^-enterobactin-Ciprofloxacin conjugate (Fe^+3^-Cnj-1) at 62 µg/ml and (**B**), Fe^+3^-enterobactin-Fosfomycin conjugate (Fe.^+3^-Cnj-2) at 1 mg/ml, compared to their free antibiotics Ciprofloxacin (CPF) and Fosfomycin (Fos), against the *E. coli* OQ866153

Figure 3A, B illustrate the time-kill kinetics of Ciprofloxacin and Fosfomycin, both individually and in combination with Fe^+3^-enterobactin, against *E. coli* OQ866153. The kinetics of the antibiotics were found to be comparable to those of the bacterial control. Interestingly, the bactericidal activity of Ciprofloxacin was significantly increased at 24 h after treatment with the addition of Fe^+3^-enterobactin. Initially, there was only a weak inhibition, but this was improved to a bacteriostatic effect. The reductions in bacterial colony-forming units (cfu/ml) at different time points were as follows: 0 h—0.9 log_10_ cfu/ml, 2 h—2.3 log_10_ cfu/ml, 4 h—2.54 log10 cfu/ml, 6 h—2.51 log_10_ cfu/ml, and 8 h—bactericidal effects. This bactericidal effect continued for 12 h and lasted up to 24 h, resulting in a reduction of 3.66 log_10_ cfu/ml (Fig. 3A). Additionally, the Fe^+3^-Cnj-2 conjugate demonstrated a bacteriostatic effect for up to 4 h after treatment, resulting in a reduction of 1.3 and 2.7 log_10_ cfu/ml after 2 and 4 h of incubation, respectively. This was followed by an increase in bactericidal activity, with a reduction of 4 to 5 and 5.7 log_10_ cfu/ml after 6, 8, and 24 h of treatment (Fig. 3B). It is worth noting that there is no prior research available on the bactericidal effects of these conjugates against antibiotic-resistant *E. coli* strains. Data analysis was conducted using Graph Pad Prism (Graph Pad Software Inc., San Diego, CA, USA) Version 9.0 for Windows, with one-way ANOVA followed by Dunnett's post hoc test. The results are presented in Tables S1 and S2.

#### A postantibiotic effect (PAE) assay

**Fig. 4:**
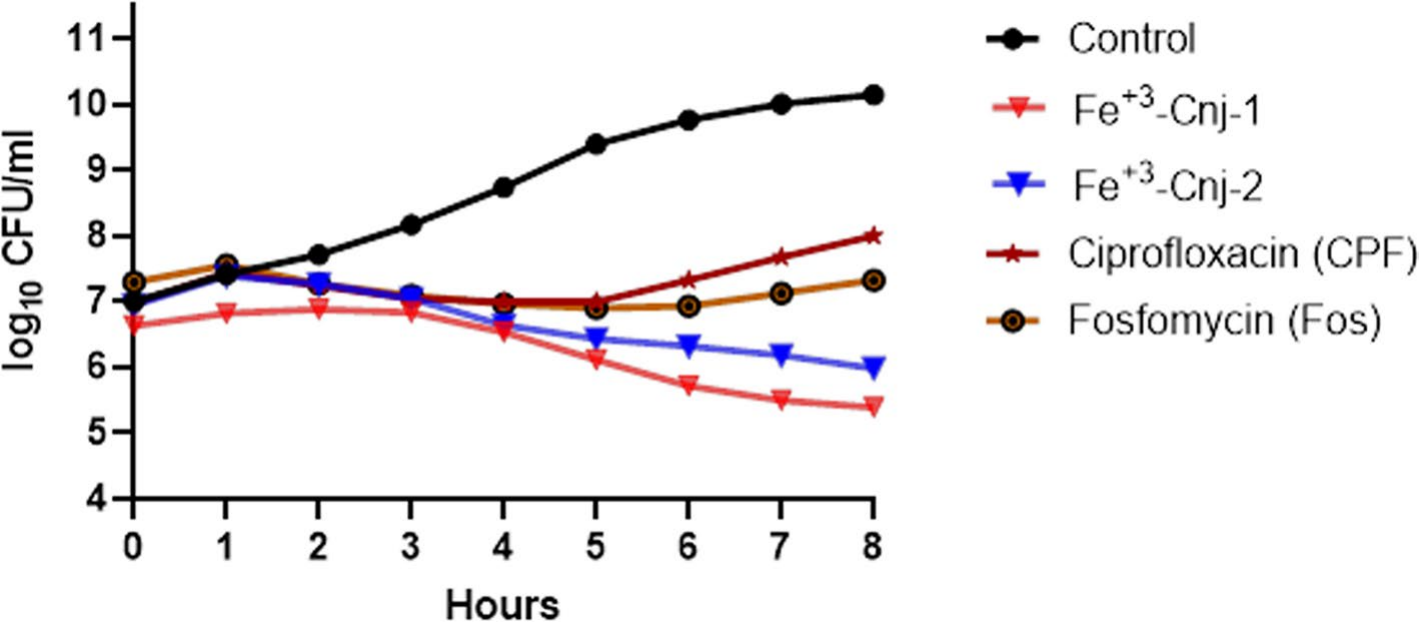
The postantibiotic effect (PAE), or persistent suppression of bacterial growth after brief exposure of a bacterial culture to an antimicrobial agent

Table 4 shows the postantibiotic effects (PAE) of Fe^+3^-Cnj-1, Fe^+3^-Cnj-2, CPF, and Fos on *E. coli*, measured as the duration of growth suppression after antibiotic removal. Fe^+3^-Cnj-1 has a PAE of 3 h, indicating that bacterial growth is inhibited for an additional 3 h after antibiotic exposure. Fe^+3^-Cnj-2 has a slightly shorter PAE of 2 h compared to Fe^+3^-Cnj-1. CPF and Fe^+3^-Cnj-2 both have a PAE of 2 h, suggesting sustained growth inhibition even after antibiotic removal. On the other hand, Fos has a PAE of 0 h, indicating that bacterial growth resumes immediately after the antibiotic is removed (Fig. 4).

#### DNA-gyrase inhibition assay

**Fig. 5:**
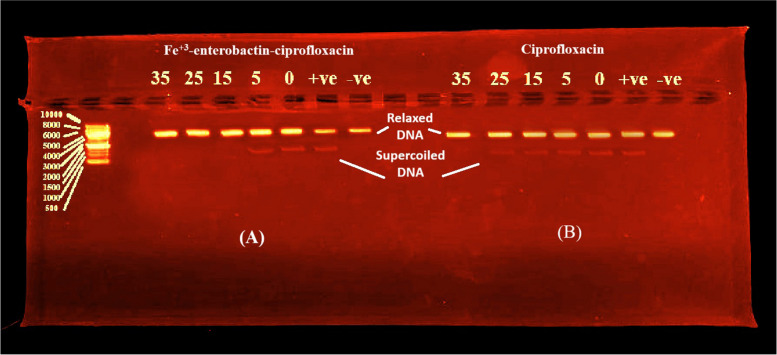
Agarose gel electrophoresis for DNA *gyr*ase assay of (**A**), Fe^+3^-enterobactin-ciprofloxacin (Fe^+3^-Cnj-1) conjugate and (**B**), Ciprofloxacin. + ve, positive control with DNA *gyr*ase and no conjugate; -ve; negative control without DNA *gyr*ase and conjugate

To assess the ability of Fe^+3^-Cnj-1 to inhibit DNA gyrase, a crucial in vitro assay was conducted. This assay is crucial for understanding the antimicrobial efficacy of Ciprofloxacin and its conjugates, as it evaluates their ability to impede the drug target DNA gyrase. The conjugate was tested at different concentrations (0, 5, 15, 25, and 35 μg/ml) and it was found that at 5 μg/ml, there was no suppression of DNA gyrase activity, as evidenced by the presence of supercoiled DNA plasmids. The conjugate showed inhibition of DNA gyrase at concentrations of 15 μg/ml and higher, eliminating supercoiled DNA plasmids at 25 μg/ml. These results demonstrate that Fe^+3^-Cnj-1 has a higher *gyrase* inhibitory activity compared to the original Ciprofloxacin drug (35 μg/ml), as shown in Fig. 5.

#### Antibiofilm quantitative assay

**Fig. 6:**
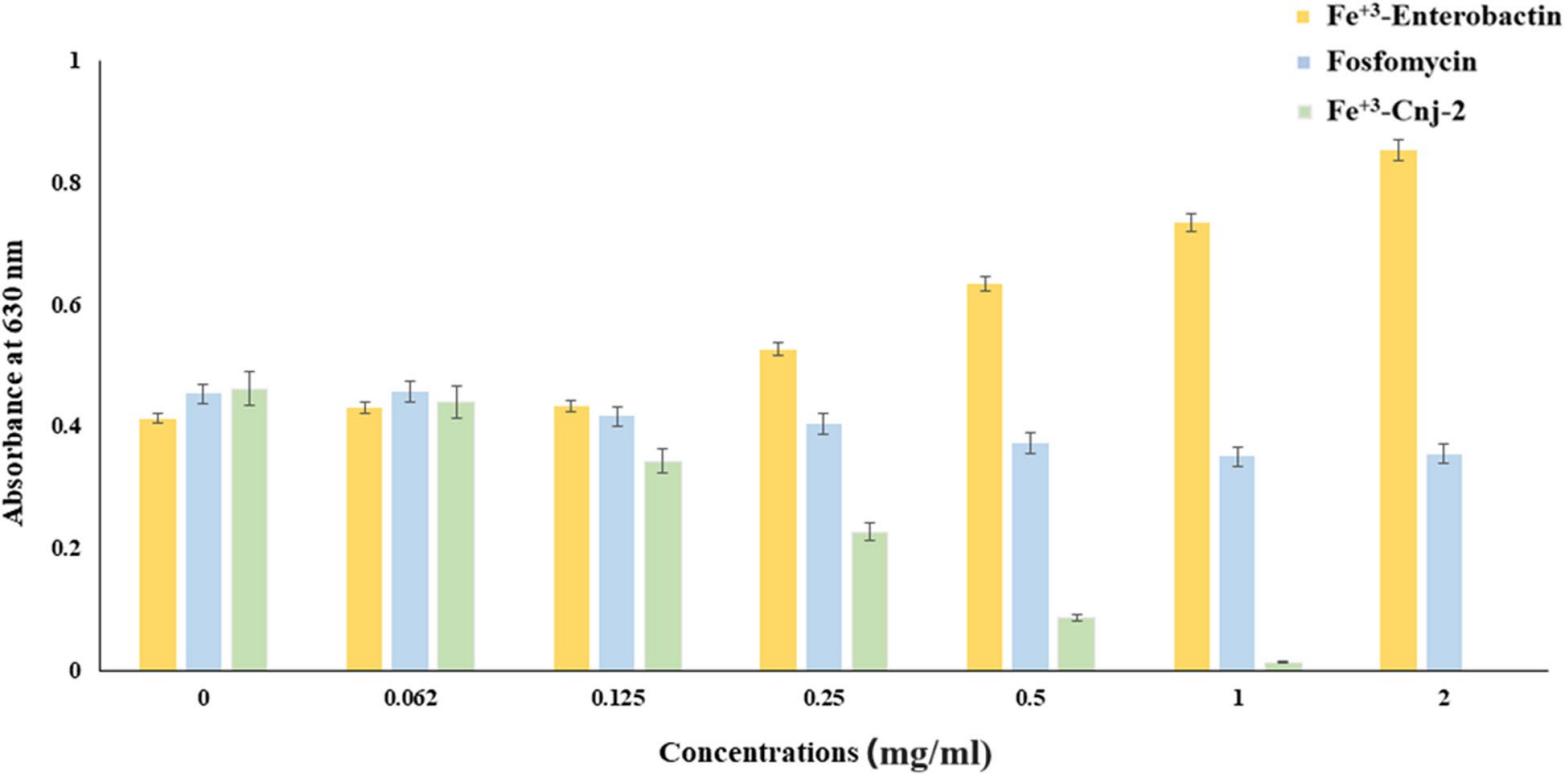
Effect of Fe^+3^-enterobactin, Fosfomycin, and Fe^+3^-enterobactin-Fosfomycin (Fe^+3^-Cnj-2) on *E. coli* OQ866153 biofilm formation

Different concentrations (0, 0.062, 0.125, 0.25, 0.5, 1, and 2 mg/ml) of Fe^+3^-enterobactin, Fosfomycin, and Fe^+3^-Cnj-2 were used to determine the level of biofilm formation by *E. coli* OQ866153. In Fig. 6, it is evident that there was a significant reduction (*p* < 0.0001) in biofilm formation as the concentration of the conjugate increased. At the MIC level of 0.5 mg/ml, there was a noticeable decrease in biofilm formation. The inhibition percentages of biofilm formation ranged from 4.7% to 100% when the conjugate was added at concentrations of 0.062 to 2 mg/ml. In comparison, Fosfomycin achieved a maximum inhibition percentage of 21.58% at a concentration of 2 mg/ml. The results revealed that the presence of Fe^+3^-enterobactin enhanced the effectiveness of Fosfomycin by facilitating its cellular uptake via enterobactin-binding proteins (*FepABCDG*) on the cell membrane, thus overcoming obstacles such as efflux proteins. However, it was observed that Fe^+3^-enterobactin alone significantly (*p* < 0.0001) increased biofilm formation, with a percentage of 106.52% at a concentration of 2 mg/ml compared to the untreated control group.

#### Environmental Scanning Electron Microscope (ESEM) analysis

**Fig. 7:**
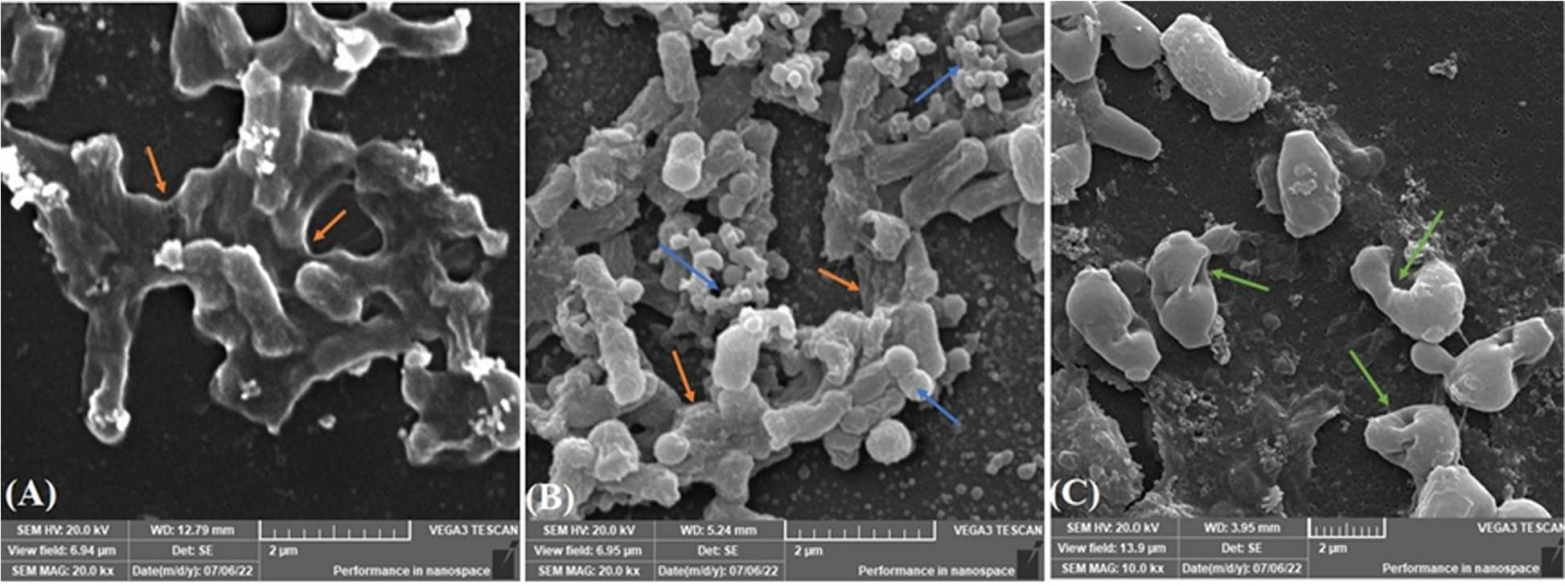
ESEM micrographs of *E. coli* OQ866153, (**A**, 20000x), untreated showing EPS of the biofilm structure (orange arrow); (**B**, 20000x) treated with Fe^+3^-enterobactin showing increased EPS matrix (orange arrow) and proliferation of cells (blue arrow), and (**C**; 10000x), treated with Fe^+3^-enterobactin-Fosfomycin (Fe.^+3^-Cnj-2) conjugate showing few and disrupted cells (green arrow)

The ESEM analysis demonstrated that Fe^+3^-Cnj-2 had an inhibitory effect on biofilm formation by *E. coli* OQ866153. In Fig. 7A, it can be observed that the untreated sample of *E. coli* OQ866153 attached to the glass surface and formed a biofilm with a dense extracellular polysaccharide (EPS) matrix containing bacterial cells. However, in Fig. 7B, the treated sample with Fe^+3^-enterobactin exhibited a higher density of an EPS matrix, which wrapped a larger number of proliferated bacterial cells compared to the untreated sample.

In contrast, when the concentration of the Fe^+3^-Cnj-2 conjugate was 2 × MIC (1 mg/ml), only a small number of cells were scattered on the glass surface, the biofilm structure had a significantly reduced amount of extracellular polymeric substance (EPS), and the integrity of some bacterial cells was compromised, as illustrated in Fig. 7C. These results suggest that the Fe^+3^-Cnj-2 conjugate can effectively hinder the formation of biofilms by *E. coli* OQ866153.

### In silico* docking studies*

#### Homology Model

The 3D quaternary structures of *FepC, FepD*, and *FepG* were constructed based on their sequence identity with the target proteins. The homology models of *FepC, FepD*, and *FepG* were analysed and found to have a 42.19%, 41.06%, and 38.89% identity, respectively, with the iron-hydroxamate import ATP-binding protein *FhuC* structure of a ferrichrome importer *FhuCDB* from *E. coli* (pdb: 7lb8), vitamin B12 import system permease protein *BtuC* ABC-transporter *BtuCD* (pdb: 2qi9), and hemin transport system permease protein *HmuU* of the bacterial heme transporter *HmuUV* from *Yersinia pestis* (pdb: 4g1u). The 3D structure of *FepC, FepD*, and *FepG* was illustrated in Fig. S3.

#### Binding Affinity interactions

**Fig. 8:**
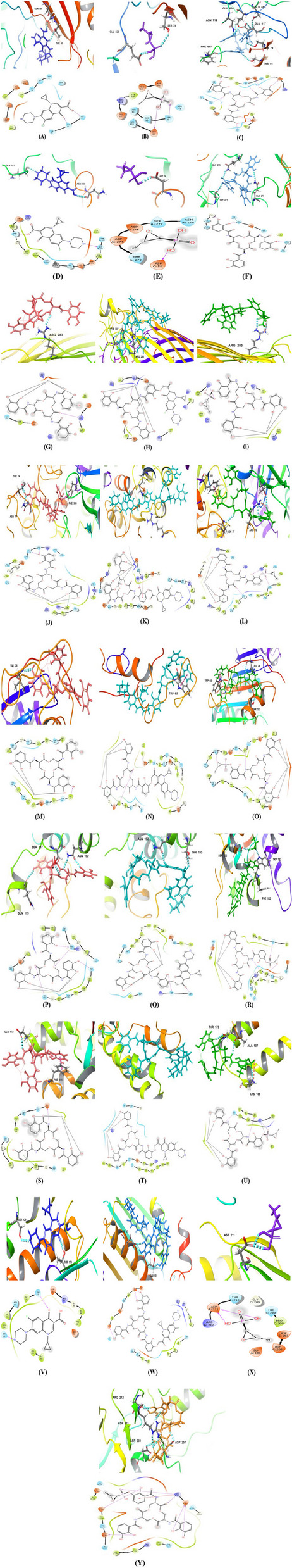
The 2D and 3D interaction between *E. coli* proteins and ligands*.***A**, *AcrB* and Ciprofloxacin; (**B**), *AcrB* and Fosfomycin; (**C**) *AcrB* and enterobactin; (**D**), *TolC* and Ciprofloxacin; (**E**), *TolC* and Fosfomycin; (**F**), *TolC* and enterobactin; (**G**), *FepA* and Fe^+3^-enterobactin; (**H**), *FepA* and Fe^+3^-enterobactin-Ciprofloxacin; (**I**), *FepA* and Fe^+3^-enterobactin-Fosfomycin; (**J**), *FepB* and Fe^+3^-enterobactin; (**K**), *FepB* and Fe^+3^-enterobactin-Ciprofloxacin; (**L**), *FepB* and Fe^+3^-enterobactin-Fosfomycin; (**M**), *FepC* and Fe^+3^-enterobactin; (**N**), *FepC* and Fe^+3^-enterobactin-Ciprofloxacin; (**O**), *FepC* and Fe^+3^-enterobactin- Fosfomycin; (**P**), *FepD* and Fe^+3^-enterobactin; (**Q**), *FepD* and Fe^+3^-enterobactin-Ciprofloxacin; (**R**), *FepD* and Fe^+3^-enterobactin-Fosfomycin; (**S**), *FepG* and Fe^+3^-enterobactin; (**T**), *FepG* and Fe^+3^-enterobactin-Ciprofloxacin; (**U**), *FepG* and Fe^+3^-enterobactin-Fosfomycin; (**V**), DNA *gyr*ase and Ciprofloxacin; (**W**), DNA *gyr*ase and Fe^+3^-enterobactin-Ciprofloxacin; (**X**), *MurA* and Fosfomycin; (**Y**), *MurA* and Fe^+3^-enterobactin- Fosfomycin

The molecular docking study of the enterobactin, Ciprofloxacin, and Fosfomycin ligands to the *E. coli* cell membrane efflux proteins *AcrB* (PDB: 1T9U) and *TolC* (PDB: 1EK9) has been shown in Table 2 and Fig. 8A-F. The docking results indicated that the enterobactin showed the highest binding affinity to protein active sites of *AcrB/TolC*, with docking scores -6.565/-6.308 kcal/mol, respectively, by forming 8 H-bonds with SER^79^, THR^91^, PHE^617^, GLU^683^, ASN^719^, GLU^817^, and GLU^826^ of *AcrB* active site, and 3-H bonds with GLN^273^ (A), GLN^273^ (C), and GLY^271^*TolC* protein active site. However, both Ciprofloxacin and Fosfomycin showed closely similar binding affinity to the protein active sites of *AcrB/TolC* with moderate scores of -4.151/-4.113 and -4.964/-4.917 kcal/mol, respectively. These indicated that the cell membrane efflux proteins *AcrB* and *TolC* contributed to the export of free iron enterobactin, Ciprofloxacin, and Fosfomycin outside the bacterial cell.

**Table 2:** The molecular docking study between ligands and the *E. coli* cell proteins

Proteins	Ligands	Docking score	Interact H-bonds	No. of H-bonds	Pi-cations	Pi-pi stacking	Salt bridges
***AcrB***	**Ciprofloxacin**	**-4.151**	**GLN**^**89**^**, THR**^**91**^	**2**			
	**Fosfomycin**	**-4.964**	**SER**^**79**^**, GLU**^**826**^	**2**			
	**Enterobactin**	**-6.565**	**SER**^**79**^**, THR**^**91**^**, PHE**^**617**^**, GLU**^**683**^**, ASN**^**719**^**, GLU**^**817**^**, GLU**^**826**^	**8**			
***TolC***	**Ciprofloxacin**	**-4.113**	**ASN**^**58**^**, GLN**^**273**^	**2**			
	**Fosfomycin**	**-4.917**	**ASP**^**56**^	**1**			
	**Enterobactin**	**-6.308**	**GLN**^**273**^** (A), GLN**^**273**^** (C), GLY**^**271**^	**3**			
***FepA***	**Fe**^**+3**^**-enterobactin**	**-5.585**	**ARG**^**283**^	**1**			
	**Fe**^**+3**^**-enterobactin -Ciprofloxacin**	**-6.3**	**ARG**^**283**^**, PHE **^**337**^	**2**			
	**Fe**^**+3**^**-enterobactin -Fosfomycin**	**-5.605**	**ARG**^**283**^	**1**			**ARG**^**283**^
***FepB***	**Fe**^**+3**^**-enterobactin**	**-4.388**	**ASN**^**77**^**, THR**^**74**^	**2**		**PHE**^**300**^	
	**Fe**^**+3**^**-enterobactin -Ciprofloxacin**	**-6.789**	**ARG**^**87**^	**1**	**LYS**^**270**^		
	**Fe**^**+3**^**-enterobactin -Fosfomycin**	**-5.986**	**ASN**^**77**^**, ARG**^**78**^**, THR**^**200**^	**3**		**PHE**^**300**^	**ARG**^**78**^
***FepC***	**Fe**^**+3**^**-enterobactin**	**-7.09**	**TRP**^**65**^	**1**		**TRP**^**65**^	
	**Fe**^**+3**^**-enterobactin -Ciprofloxacin**	**-7.120**	**TRP**^**65**^**, LEU**^**26**^**, THR**^**52**^	**3**		**TRP**^**65**^	
	**Fe**^**+3**^**-enterobactin -Fosfomycin**	**-7.675**	**VAL**^**28**^	**1**			
***FepD***	**Fe**^**+3**^**-enterobactin**	**-6.217**	**GLN**^**179**^**, SER**^**187**^**, ASN**^**192**^	**4**			
	**Fe**^**+3**^**-enterobactin -Ciprofloxacin**	**-6.810**	**ASN 192, THR 195**	**2**			
	**Fe**^**+3**^**-enterobactin -Fosfomycin**	**-8.016**	**SER**^**102**^**, PHE**^**182**^**, TRP**^**183**^	**3**			
***FepG***	**Fe**^**+3**^**-enterobactin**	**-5.318**	**GLU**^**172**^			**PHE**^**111**^	
	**Fe**^**+3**^**-enterobactin -Ciprofloxacin**	**-6.360**	**No Bonds**				
	**Fe**^**+3**^**-enterobactin -Fosfomycin**	**-6.3136**	**THR**^**173**^**, ALA**^**107**^**, LYS**^**168**^				**LYS**^**168**^
**DNA *gyr*ase**	**Ciprofloxacin**	**-6.264**	**SER**^**129**^**, THR**^**173**^	**2**			
	**Fe**^**+3**^**-enterobactin -Ciprofloxacin**	**-8.597**	**GLU**^**50**^**, ASP**^**57**^	**3**			
***MurA***	**Fosfomycin**	**-3.756**	**ASP**^**211**^	**2**			
	**Fe**^**+3**^**-enterobactin -Fosfomycin**	**-5.481**	**ASP**^**260**^**, ASP**^**257**^**, ARG**^**212**^**, ASP**^**211**^	**7**	**ARG**^**212**^		

*ALA* Alanine, *ARG* Arginine, *ASN* Asparagine, *ASP* Aspartic acid, *GLN* Glutamine, *GLU* Glutamic acid, *GLY* Glycine, *LEU* Leucine, *LYS* Lysine, *PHE* Phenylalanine, *SER* Serine, *THR* Threonine, *TRP* Tryptophan, *VAL* Valine

Additionally, the Fe^+3^-Cnj-1/Cnj-2 conjugates exhibited the strongest affinity to enterobactin binding proteins *FepABCDG*, with respective docking scores of -6.3/-5.605, -6.789/-5.986, -7.120/-7.675, -6.810/-8.016, and -6.360/-6.3136 kcal/mol. Fe^+3^-Enterobactin followed closely with docking scores of -5.585, -4.388, -7.09, -6.217, and -5.318 kcal/mol, respectively (Table 2 and Fig. 8G-U). Notably, the conjugates of enterobactin with Ciprofloxacin and Fosfomycin exhibited superior binding affinity towards the *E. coli* DNA supercoiling protein-DNA *gyr*ase and *MurA*, as indicated by their docking scores (-8.597 and -5.481, respectively), compared to that of the native antibiotics were comparatively lower, with scores of -6.264 and -3.756, respectively (Table 2 and Fig. 8V-Y). These findings suggested that Fe^+3^-enterobactin could enhance the efficacy of Fosfomycin and Ciprofloxacin by enabling their entry into the cells via *FepABCDG* proteins on the cell membrane, circumventing the *AcrB* and *TolC* membrane barriers.

### Assessment of cytotoxic effects on HEK293 human embryonic kidney cells through MTT assay

**Fig. 9:**
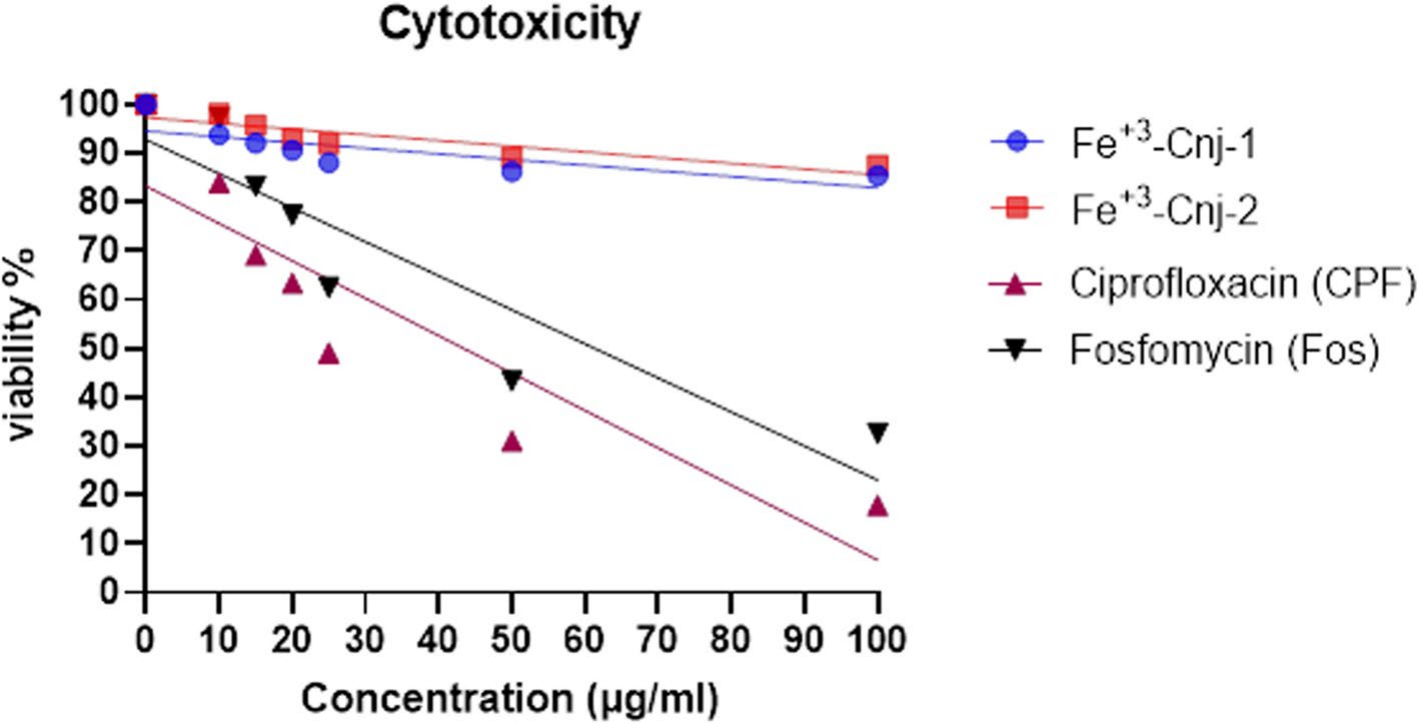
Concentration–Response Relationship for Fe^+3^-Cnj-1, Fe^+3^-Cnj-2, CPF, and Fos on Cell Viability in HEK293 Human Embryonic Kidney Cells

Table 3 shows the IC50 values for Fe^+3^-Cnj-1, Fe^+3^-Cnj-2, CPF, and Fos compounds on HEK293 human embryonic kidney cells obtained from the cell viability assay. Fe^+3^-Cnj-1 had an IC50 of 553.2 μg/ml, while Fe^+3^-Cnj-2 had an IC50 of 385.7 μg/ml. In comparison, the IC50 values for ciprofloxacin and Fosfomycin were 23.8 μg/ml and 41.4 μg/ml, respectively (Fig. 9).

**Table 3:** IC50 values of Fe^+3^-Cnj-1, Fe^+3^-Cnj-2, CPF, and Fos in HEK293 Human Embryonic Kidney Cells Assessed by MTT Assay

Compound	Predicted IC50 (μg/ml)
Fe^+3^-Cnj-1	553.1977
Fe^+3^-Cnj-2	385.6618
Ciprofloxacin (CPF)	23.79808
Fosfomycin (Fos)	41.35488

## Discussion

Microbial siderophores have many applications in the environmental and medical sciences. In the environmental field, various types of siderophores have been found to act as agents for biological control against phytopathogens, improving the growth of several plant species, detoxifying samples contaminated with heavy metals (making them useful in bioremediation), and serving as biosensors [19–21]. In the medical field, siderophores can be utilised to get rid of transuranic elements from the body, treat sickle cell anaemia (iron overload), treat malaria, and have anticancer potential activity [22]. In addition, siderophores use the "Trojan horse strategy" to form complexes with antibiotics, enabling selective delivery of antibiotics to antibiotic-resistant bacteria through iron uptake machinery on the outer membrane to overcome the effective permeation barrier of cell envelope [18]. In this study, enterobactin, a mixed ligand of the extensively drug resistant (XDR) *E. coli* OQ866153, was conjugated with Ciprofloxacin and Fosfomycin individually to aid their active absorption via particular enterobactin binding receptors *FepABCDG*, bypass the typical permeability challenge caused by efflux proteins *AcrB* and *TolC*, and thereby enhancing their effectiveness against the tested *E. coli* strain.

Active transport is necessary for the recognition and incorporation of ferric enterobactin complexes due to their low concentration and large size. Particularly, the process of ferric enterobactin recognition starts by the outer membrane receptor *Fep*A, which is then transported into the periplasm with energy provided by a TonB-ExbB-ExbD complex [23]. After crossing the outer membrane, the ferric enterobactin is translocated into the cytoplasm through the ATP binding cassette transporter involves binding of the ferric enterobactin by the periplasmic binding protein *FepB*, which is more specific to enterobactin than other similar siderophores, followed by interaction with the cytoplasmic membrane spanning proteins *FepD* and *FepG* [24]. Finally, ATP hydrolysis via the membrane-bound ATP binding protein *FepC* transports the ferric enterobactin across the cytoplasmic membrane [25, 26]. This specific transport machinery had been investigated in this study through the in vitro detection of enetrobactin receptors *FepABCDG* and in silico study that exhibited high binding affinity of Fe^+3^-enterobactin (-5.59, -4.39, -7.09, -6.23, and -5.318), Fe^+3^-Cnj-1 (-6.3, -6.79, -7.12, -6.81, and -6.36), and Fe^+3^-Cnj-2 (-5.61, -5.99, -7.68, -8.02, and -6.61) to *FepABCDG* proteins, respectively. This agreed with Neumann et al. (2018), who reported that the alkyl-linked enterobactin-Ciprofloxacin conjugate was transported into the cytoplasm of *E. coli* via the enterobactin-specific *FepABCDG* proteins, and any mutation in the inner membrane *FepCDG* transporter revoked this conjugate antibacterial activity [25]. In the cytoplasm, iron release involves two possible ways: the first one depends on iron reductase for the reduction of Fe^+3^-enterobactin to free Fe^+2^, whereas the second involves hydrolysis of Fe^+3^-enterobactin. According to Bleuel et al. (2005), the export of iron free enterobactin across the outer membrane in *E. coli* did not require any of the seven-resistance nodulation cell division (RND) proteins *CusA, AcrB, AcrD, AcrF, MdtF (YhiV) or MdtBC (YegNO)*, but relied on the outer membrane channel tunnel protein TolC [27]. In contrast, Pham et al. 2019 reported that the *ArcB*, *ArcD*, or *MdtABC* (RND-class) inner membrane efflux pumps and the outer membrane *TolC* efflux protein iterate enterobactin out of the cell [26]. These findings were consistent with Guest et al. (2019), who reported that *E. coli TolC* mutants starved for iron, synthesis but cannot secrete enterobactin, leading to its accumulation in the periplasm [28]. Additionally, Chowdhury et al. (2019) and Shi et al. (2019) revealed that *AcrAB*-*TolC*, an efflux pump expressed constitutively in *E. coli*, is a natural mechanism of multidrug resistance, including resistance to antibiotics such as chloramphenicol, fluoroquinolone, tetracycline, novobiocin, fusidic acid, nalidixic acid, and β-lactams, as well as to dyes, detergents, and most lipophilic antibiotics [29, 30]. In a prior investigation Khazaal et al. (2022), it was observed that the *E. coli* 49 (*E. coli* OQ866153) strain demonstrated resistance to all antibiotics tested, except for kanamycin [31]. Furthermore, in the current study, it was discovered that this strain was also resistant to Ciprofloxacin and Fosfomycin antibiotics. The extensive level of antibiotic resistance observed in *E. coli* OQ866153 may be attributed to the presence of *AcrB-TolC* efflux genes detected in this study. This result was found to be consistent with [30], who reported that all tested multidrug resistant (MDR) *E. coli* encoded the efflux pump genes *AcrAB-TolC*, which might be contributed to their antibiotic resistance.

The global crisis of antibiotic-resistant microbial infections necessitates the development of new antibiotics. A promising strategy for expanding the antibiotic repertoire is drug repurposing. Therefore, this study repurposed the targeted antibiotics Ciprofloxacin (bacterial DNA *gyr*ase and replication inhibitory) and Fosfomycin (bacterial *MurA* and biofilm inhibitory) by conjugating to enterobactin, the tricatecholate siderophore synthesis by Enterobacteriaceae to chelate iron. To date, it has been proven that the most effective siderophore-antibiotic conjugates were those delivered by TonB-dependent outer membrane transporters, such as ferric enterobactin (*FepA*), iron catecholate (*CirA*), and ferric iron uptake-(*Fiu*), to the periplasmic space [32]. The results revealed that 24 h post-treatment, the efficacy of Ciprofloxacin and Fosfomycin against the *E. coli* OQ866153 was enhanced by Fe^+3^-enterobactin. Of great significance, the Fe^+3^-Cnj-1 (MIC = 31.25 µg/ml) and Fe^+3^-Cnj-2 (MIC = 500 µg/ml) conjugates demonstrated bactericidal properties leading to a decline of 3.66 and 5.7 log10 cfu/ml, respectively, in bacterial count and persisted for 24 h of treatment. This antibacterial action was explained by Neumann et al. (2018), who reported that in *E. coli* strains, the alkyl-linked enterobactin-Ciprofloxacin conjugate was transported via *FepABCDG* into the cytoplasm where it must be hydrolyzed by the salmochelin (C-glycosylated enterobactin) hydrolase *IroD* (only expressed by pathogens harbouring the *IroA* gene cluster, *IroBCDEN*) to exert its antibacterial activity against the pathogenic *E. coli* CFT073 and the non-pathogenic K12 one that complemented with *IroD,* while the enterobactin hydrolase *Fes* was not sufficient to perform this function [25]. Similarly, Guo et al. (2023), reported that the enterobactin-platinum (Pt (IV)) prodrug of cisplatin conjugate (l-EP) and its enantiomer (d-EP) enhanced the antibacterial activity of cisplatin by causing filamentation and inhibition of *E. coli* k12 and CFT073 strains [33]. According to Negash et al. (2019), the combination of enterobactin mimic and amoxicillin resulted in improved MICs effects against the Gram-negative bacteria, particularly *P. aeruginosa* KW799, with a value of 0.05 µM, compared to *E. coli* ATCC 25,922 (1.56 µM) and *Klebsiella pneumoniae* ATCC 8303 (× 68 ˃ 100 µM) [34]. In contrast, Fardeau et al. (2014) synthesized the same enterobactin mimic and conjugated to Ciprofloxacin. However, the antibacterial activity against *P. aeruginosa* was moderate, and the MIC was not significantly reduced compared to Ciprofloxacin alone [35]. This finding was found to be consistent with Neumann et al. (2018), who reported that the MIC values (0.1–1 µM) of the enterobactin-Ciprofloxacin alkyl-linked conjugate were similar to those of the parent drug against the *E. coli* strains UTI89 and CFT073 [25]. Stimulatingly, Ampicillin conjugated with natural enterobactin by Zheng and Nolan (2014) displayed a noteworthy 1000-fold decrease in MIC as compared to ampicillin in *E. coli* strains [36]. It also exhibited selective killing of *E. coli*, even in the presence of Gram-positive *S. aureus* during co-culture. As reported by [34, 37], the biscatecholate-monohydroxamate mixed ligand-carbacephalosporin conjugate demonstrated an MIC of 7.80 nM against *Acinetobacter baumannii*, which was considerably lower than the parent antibiotic with an MIC greater than 128 µM. The conjugate exhibited moderate antibacterial activity against *S. aureus* (MIC = 32 µM) and *E. coli* (8 µM). Interestingly, Ghosh et al. (2017) combined daptomycin, an antibiotic effective only against Gram-positive bacteria, with a mixed ligand analogue of fimsbactin, a selective siderophore found in *Acinetobacter baumannii* [38]. The resulting conjugate was discovered to be highly potent against various strains of Gram-negative *A. baumannii*, including those that were resistant to multiple drugs, and was specifically identified and effective both in vitro and in vivo. This demonstrated that utilization of the sideromycin Trojan horse strategy showcases the potential to expand the strong efficacy of a typically Gram-positive exclusive antibiotic to produce a formidable Gram-negative antibiotic, even if the drug or warhead is larger than the transporting siderophore component. This was consistent with the findings of Liu et al. (2018) who reported that oxazolidinone Gram-positive antibiotics were active against Gram-negative bacteria (clinical isolates of *Acinetobacter baumannii* as well as strains producing high levels of ADC-1 β-lactamase) when efficiently delivered into bacteria using a Trojan-horse strategy and cephalosporin as a hydrolysable linker [39]. This strategy could be applied to any Gram-positive antibiotic or drug to efficiently deliver it to even highly resistant bacteria, pathogens of major concern around the world [40]. These results indicated how utilizing natural siderophores can result in effective conjugates.

The postantibiotic effect (PAE) assay was conducted to evaluate the sustained growth suppression of Fe^+3^-Cnj-1, Fe^+3^-Cnj-2, ciprofloxacin (CPF), and fosfomycin (Fos) on *E. coli* after the removal of antibiotics. The results, presented in Table 4, demonstrated the duration of growth inhibition for each compound.

**Table 4:** Postantibiotic Effects on E. coli Antibiotic PAE (hrs) Fe^+3^-Cnj-1, 3 Fe^+3^-Cnj-2, CPF, and Fos

Antibiotic	PAE (hrs)
Fe^+3^-Cnj-1	3
Fe^+3^-Cnj-2	2
CPF (Ciprofloxacin)	2
Fos (Fosfomycin)	0

Fe^+3^-Cnj-1 exhibited a PAE of 3 h, indicating that bacterial growth remained suppressed for an additional 3 h following antibiotic exposure. Fe^+3^-Cnj-2, on the other hand, had a slightly shorter PAE of 2 h compared to Fe^+3^-Cnj-1. Both CPF and Fe^+3^-Cnj-2 displayed a PAE of 2 h, indicating sustained growth inhibition even after the antibiotics were removed. In contrast, Fos exhibited a PAE of 0 h, suggesting that bacterial growth immediately resumed once the antibiotic was eliminated.

These findings highlight the varying effects of the tested compounds on the post-antibiotic growth kinetics of *E. coli*. Fe^+3^-Cnj-1 demonstrated a longer-lasting impact, with sustained growth suppression for an additional 3 h beyond the antibiotic exposure period. Fe^+3^-Cnj-2, CPF, and Fe^+3^-Cnj-2 also exhibited notable PAE, albeit slightly shorter in duration at 2 h. Interestingly, Fos did not exhibit any significant PAE, indicating a lack of sustained growth suppression in *E. coli* following antibiotic removal.

The observed PAE values underline the potential of Fe^+3^-Cnj-1, Fe^+3^-Cnj-2, and CPF as effective agents for prolonged growth inhibition in *E. coli* infections, while highlighting the limited PAE of Fos. These findings contribute to our understanding of the dynamics of bacterial growth and the potential efficacy of these compounds in clinical settings.

To investigate the impact of Fe^+3^-Cnj-1 conjugate to inhibit DNA-*gyr*ase, an in vitro assay was carried out using a commercial DNA *gyr*ase supercoiling assay. In this study, the conjugate exhibited a complete absence of supercoiled DNA plasmids due to inhibition of the *gyr*ase enzyme at 25 μg/ml in comparison with the parent drug (35 μg/ml), indicating an increase in *gyr*ase inhibitory activity of the conjugate compared to the Ciprofloxacin drug with docking scores of -8.597 and -6.264 kcal/mol, respectively. This result agreed with Lamut et al. (2020), who discovered that the 4,5,6,7-tetrahydrobenzo(*d*)thiazole derivatives conjugated with catechol siderophore mimic moiety, were more potent *E. coli* DNA *gyr*ase inhibitors (with IC_50_ of 0.058 μM) than the positive control novobiocin (IC50, 0.17 μM) [41]. In contrast, Neumann et al. (2018) reported that the enterochelin-Ciprofloxacin alky-linked conjugate and DHBS-Ciprofloxacin conjugate exhibited DNA *gyr*ase inhibitory activity with IC_50_ equal to 70 and 20 μM, lower than that of the unmodified parent drug (IC_50_: 0.25 μM). Additionally, Sanderson et al. (2020) demonstrated that the DNA *gyr*ase inhibitory activity of Salmochelin S4-Ciprofloxacin conjugate (75 μM) was significantly lower than that of the parent Ciprofloxacin (10 μM). This was attributed to the obstacle of delivering the conjugate through Fe^+3^-siderophore transporters into the bacterial cell [42].

The uptake of iron siderophore may have a significant impact on the formation of microbial biofilm [43]. For instance, the formation of biofilm by *P. aeruginosa* is dependent on the transport of the siderophore pyoverdine [43]. Consequently, siderophore antibiotics may have unique antimicrobial properties during treatment of biofilm [44]. In this study, we compared the in vitro activities of Fe^+3^-enterobactin, Fe^+3^-Cnj-2 conjugate and comparator antibiotic Fosfomycin against the biofilm formation via *E. coli* OQ866153. The results indicated that at a concentration of 2 mg/ml, the Fe^+3^-Cnj-2 conjugate exhibited a complete inhibition (100%) of biofilm formation, whereas Fosfomycin only showed a 21.58% inhibition. This may be attributed to the binding affinity of the conjugate with the protein active site of *E. coli MurA* was higher than that of Fosfomycin alone, as evidenced by docking scores of -5.481 and -3.756 kcal/mol, respectively. These findings suggested that Fe^+3^-enterobactin enhances the effectiveness of Fosfomycin by facilitating its entry into the cells through enterobactin binding proteins *FepABCDG* on the cell membrane, thereby avoiding membrane barriers such as efflux proteins. This agreed with Negash et al. (2019) who reported that, to overcome cell permeability obstacles, β-lactam or Ciprofloxacin antibiotics conjugated to siderophores transfer through active iron transporters and could alter the cell wall or inhibit DNA *gyr*ase [34]. Consistently, Pybus et al. (2021), reported that the cefiderocol (siderophore cephalosporin utilizes TonB-dependent iron transporters) displayed a 93% reduction in *P. aeruginosa* biofilm superior to 49–82% reduction obtained by comparator antibiotics. Whereas, against *E. coli* and *A. baumanni* biofilm, imipenem was found to be the most potent with ˃ 90% reduction compared to 67–80% of cefiderocol, however, statistically the difference was not significant [44]. In contrast, Fe^+3^-Enterochelin alone significantly increased (*p* < 0.0001) the biofilm formation with percentage reached 106.52% at concentration of 2 mg/ml compared to control (untreated) one. That is attributed to, iron is crucial for mature biofilm formation and biofilm matrix stability [45]. Further examination through ESEM analysis revealed that the treated *E. coli* OQ866153 with Fe^+3^-Enterochelin was highly dense with an EPS matrix wrapped around a larger number of proliferated bacterial cells compared to the untreated one. Whereas only a few cells were strewn on the glass field, the biofilm EPS structure was completely absent, and some of the bacterial cells’ integrity was disrupted in the cells treated with Fe^+3^-enterochelin-Fosfomycin conjugate. These findings were consistent with previous researches, which found that the Fosfomycin’s bactericidal activity is caused by its binding to the *MurA* transferase protein active site, rendering it inactive and inhibiting the synthesis of UDP-N-acetylglucosamine-enolpyruvate from UDP-N-acetylglucosamine and phosphoenolpyruvate, disrupting the initial step of bacterial cell wall synthesis [46], shatter up biofilm [47], and eventually leading to the bacterial cell's destruction [48]. These results concluded that Fe^+3^-enterochelin-Fosfomycin conjugate was a potent inhibitor capable of completely inhibiting biofilm formation by the clinical pathogen *E. coli* OQ866153. No previous research has examined the bactericidal effects of this combination on antimicrobial-resistant strains of *E. coli*.

The results support the successful application of the Trojan Horse approach to antibiotics, where the conjugate is delivered into bacterial cells using siderophore transporters, specifically those with high activity such as *FepABCDG*, to achieve specific intracellular drug delivery.

The cytotoxicity of the newly developed coordination compounds Fe^+3^-Cnj-1 and Fe^+3^-Cnj-2 in HEK293 human embryonic kidney cells was evaluated and compared to the conventional antibiotic’s ciprofloxacin and fosfomycin. The results demonstrated that the coordination compounds had significantly higher IC50 values than the antibiotics, indicating lower cytotoxicity. The IC50 values for Fe^+3^-Cnj-1 and Fe^+3^-Cnj-2 were determined as 553.2 μg/ml and 385.7 μg/ml, respectively. In contrast, ciprofloxacin and fosfomycin exhibited IC50 values of 23.8 μg/ml and 41.4 μg/ml, respectively. The lower cytotoxicity of the coordination compounds in comparison to these commonly used antibiotics may be attributed to differences in their modes of action, suggesting their potential for therapeutic applications.

## Conclusion

The study investigated the use of a "Trojan Horse" strategy to overcome drug resistance in antibiotic-resistant *E. coli*. The strategy involved using enterobactin conjugated with Ciprofloxacin and Fosfomycin to deliver the drugs into bacterial cells via siderophore transporters. This approach aimed to bypass the permeability issues caused by efflux proteins. The results of the study showed that the enterobactin conjugates effectively inhibit the DNA gyrase enzyme and biofilm formation. They also had lower minimum inhibitory concentrations compared to the parent drugs. This suggests that the "Trojan Horse" strategy using enterobactin conjugates can effectively overcome permeability issues and enhance the bactericidal activity of Ciprofloxacin and Fosfomycin against antibiotic-resistant strains of *E. coli*. The study highlights the potential of using the bacterial iron uptake system to deliver drugs and overcome drug resistance mechanisms. This approach has the potential to be a promising therapeutic strategy against multidrug-resistant bacterial infections. Further research and development in this area could lead to the development of innovative therapeutic strategies to tackle the growing problem of antimicrobial resistance.

## Methods

### Source of enterobactin

Enterobactin used in this study was obtained from the clinical strain *E. coli* OQ866153, that was proven to be antibiotic resistant and siderophores producer [31]. The produced enterobactin was statistically optimized based on Plackett–Burman design (PBD) and response surface methodology (RSM) via central composite design (CCD), purified, chemically characterized using ^1^H, ^13^C NMR, IR spectroscopy, and its biosynthetic genes *EntABCDEF* was also investigated (Data under publication).

### Detection of enterobactin binding, cell membrane efflux proteins, DNA gyrase, and *MurA* genes

OLIGO 7.57 software (Molecular Biology Insights, Inc) was utilized to generate primers for genes encoding enterobactin binding proteins (*FepA*, *FepB*, *FepC*, *FepD*, and *FepG*), cell membrane efflux proteins (*AcrB* and *TolC*), DNA *gyr*ase, and *MurA*, using the sequence of *E. coli* strain 58–3 chromosome (NZ_CP050036) as a reference (Table 1). The QIAamp DNA Mini Kit (QIAGEN) was employed to extract the entire genomic DNA as directed by the manufacturer, which was then utilized as a guide for the PCR reaction. The amplified PCR samples were electrophoresed in a 1% agarose gel for 1 h at 100 V and photographed under UV transilluminator.

### Trojan-horse strategy

#### Preparation of enterobactin-antibiotic conjugates

Enterobactin-Ciprofloxacin conjugate (Cnj-1) was prepared by coupling enterobactin directly to commercially available Ciprofloxacin as described by Ghosh et al. (2017); Neumann et al. (2018); with some modifications. 10 mg of enterobactin were mixed with 5 mg of Ciprofloxacin in a 50-ml Erlenmeyer flask containing 10 ml of deionized water until all components had dissolved [25]. This process was repeated 2 times more and the pH was adjusted at 4, 7, and 9, respectively, using 20% NaOH and HCL. The solutions were stirred at 1200 rpm for 6 h at 40 °C, then left to dry in a desiccator. This method was also employed for the preparation of the enterobactin-Fosfomycin conjugate (Cnj-2). Finally, an equimolar amount of each conjugate was combined individually with FeCl_3_.6 H_2_O in water at a ratio of 1:1 v/v, to prepare Fe^+3^ complex of enterobactin conjugates (Fe^+3^-Cnj-1 and Fe^+3^-Cnj-2). The final prepared conjugates were analyzed for their purity and structure using IR-spectroscopy.

#### Fourier Transform Infrared (FTIR) Spectroscopy

The IR spectrum of enterobactin conjugates was performed using the PerkinElmer L1600400 FTIR spectrum (UK) within a wavelength range (400–4000 cm^−1^) and a resolution of 4 cm^−1^, at the central lab, Faculty of Science, Helwan University, Cairo, Egypt.

### In Vitro antibacterial studies

#### Determination of minimum inhibitory concentrations (MICs) by broth microdilution assay

The broth microdilution assay was conducted based on the protocols outlined by Balouiri et al. (2016) and Hamada et al. (2022). To prepare the stock solutions, 1 mg of each Fe^+3^-Cnj-1 and Fe^+3^-Cnj-2 conjugate was dissolved individually in 1 ml of sterile deionized water and subsequently diluted to 1/10 in sterile Mueller–Hinton broth (MHB) [49, 50]. Each well (2 to 12) in the microtiter plates received 100 µl of sterile MHB, and the first column of the plates was loaded with 150 µl of each 1/10 diluted solution. A two-fold dilution was performed by transferring 100 µl from the first well to the 11th well. Each well received 50 µl of *E. coli* OQ866153 suspension containing 1 × 10^5^ cfu/ml (OD = 0.08–0.12 at 625) except the last, which served as a blank. After incubation at 37 °C/24 h, the results were scrutinized using a ChroMate® ELISA Reader (USA) at 600 nm.

#### Time kills assay

Time kill curve analysis was performed using the techniques described by Foerster et al. (2016). An *E. coli* OQ866153 concentration of 1 × 10^5^ cfu/ml in 50 ml MHB was prepared by incubation at 37 °C and 150 rpm for 4 h [51]. Subsequently, 5 ml of each conjugate (2 × MIC) was introduced to each flask containing pre-incubated bacteria. At different time (0, 2, 4, 6, 8, and 24 h, at 37 °C) intervals, aliquots were taken and serially diluted. 100 µl of each dilution was spread on Mueller–Hinton agar (MHA) and incubated at 37 °C for 24 h, with both positive and negative controls using inoculated and un-inoculated MHB. Throughout the initial 6 h time-kill experiments, the rate of bacterial growth was assessed by monitoring variations in the number of viable bacteria (cfu/ml). Data analysis entailed calculating the average colony counts (Log10 cfu/ml) derived from three replicates of each dilution at every time interval. The quantification limit was set at 2 Log10 cfu/ml, and bacteriostatic and bactericidal effects were defined by reductions of 2 and 3 Log10 cfu/ml, respectively, compared to the initial inoculum.

#### A postantibiotic effect (PAE) assay

To assess the impact of Fe^+3^-Cnj-1, Fe^+3^-Cnj-2, ciprofloxacin (CPF), and fosfomycin (Fos) on the growth kinetics of *E. coli*, we conducted a post-antibiotic effect (PAE) assay. In this assay, *E. coli* cultures with an initial concentration of 1 × 10^6^ CFU/ml were exposed to antibiotic concentrations equivalent to 2 times the minimum inhibitory concentration (MIC) values for 2 h at 37 °C. After 2 h of exposure to the antibiotics and conjugates, the antibiotics were removed, and the cultures were transferred to fresh media. The CFU/ml was measured every hour for a total of 8 h [52]. Untreated control samples with the same initial inoculum were also included. To determine the PAE, we used the following calculation: PAE = T—C Where T = time required for count in treated culture to increase 1 log10 above count observed immediately after drug removal.

And C = corresponding time for untreated control.

#### DNA-gyrase inhibition assay

In order to assess the inhibitory effect of Fe^+3^-Cnj-1 on DNA gyrase, we conducted an in vitro assay utilizing a commercially available DNA gyrase supercoiling assay. The assay protocol followed the guidelines outlined by Maxwell et al. (2006) and Sanderson et al. (2020) [42, 53].

On ice, different concentrations (0, 5, 15, 25, and 35 μg/ml) of the inhibitors (Fe-Cnj-1 and Ciprofloxacin, in 2 μl for each) in 1.5-mL Eppendorf tubes were mixed with 24 μl reaction mixture consisting of 6 μl of 5 × assay buffer (35 mM Tris–HCl (pH 7.5), 4 mM MgCl_2_, 24 mM KCl, 2 mM DTT, 1 mM ATP, 6.5% (w/v) glycerol, 1.8 mM spermidine, 0.1 mg/ml albumin, relaxed pBR322 plasmid (0.5 μl), and water (17.5 μl). Negative control sample (tube 1) was prepared by mixing 3 μL water with 3 μl dilution buffer (100 mM KCl, 50 mM Tris–HCl (pH 7.5), 1 mM EDTA, 2 mM DTT, and 50% (w/v) glycerol). For the positive control sample (Tube 2), 3 μl water and 3 μL of diluted *E. coli gyr*ase in dilution buffer were added. The remaining tubes were treated with 3 μL of diluted *E. coli gyr*ase, followed by gentle vortexing and incubation at 37 °C for 30 min. Reactions were stopped by adding 30 μl of STEB (10 mM EDTA, 40% (w/v) sucrose, 0.5 mg/ml Bromophenol Blue, 100 mM Tris–HCl (pH 8)) and 30 μl of (v:v, 24:1) chloroform/isoamyl alcohol. After 5 s of vortexing and centrifugation for 1 min, 20 μL of the upper blue aqueous phase containing DNA products were electrophoresed in 1% agarose gel at 85 v for approximately 2 h. The gel was then stained with 1 µg/ml ethidium bromide (EtBr) in water (15 min), followed by destaining in water (5–10 min), and finally visualized using a gel documentation system.

#### Antibiofilm quantitative assay

The antibiofilm activity of Fe^+3^-Cnj-2 was determined according to Kang et al. (2019) and et al. (2022) methods with some modifications [50, 54]. 100 μl of *E. coli* OQ866153 (1 × 10^5^ cfu/ml) were added to different concentrations (0, 0.062, 0.125, 0.25, 0.5, 1, and 2 mg/ml) of Fe^+3^-Cnj-2 and Fosfomycin in a 96-well polystyrene plate and statically incubated at 37 °C for 48 h. Following incubation, sterile phosphate-buffered saline (PBS 1X, pH 7.3) was used to wash and remove the bacterial cells. The wells were then air-dried, fixed with methanol for 15 min, stained with 1% crystal violet solution for 10 min. Finally, absolute ethanol (EtOH) was loaded to all wells and examined spectrophotometrically via a microplate reader (ChroMate 4300, USA) at 630 nm. The results were expressed as the inhibition percentage of biofilm formation [55]. The significance (*p* < 0.05) was statistically calculated using ordinary one-way ANOVA via the GraphPad Prism software version 9.1.2.

#### Environmental Scanning Electron Microscope (ESEM) analysis

In a 96-well polystyrene plate, 200 μl of *E. coli* OQ866153 suspension prepared in LB broth (1 × 10^5^ cfu/ml) was inoculated, along with a glass coverslip measuring 4 mm × 4 mm. The plate was then treated with 200 μl of 1 mg/ml Fe^+3^-Cnj-2 and enterobactin, individually, and incubated for 48 h at 37 °C. A positive control was included, consisting of 200 μl of bacterial suspension and 200 μl of LB broth medium. Following incubation, the bacterial suspension was discarded and the coverslips in each well were washed three times with sterile PBS (1X, pH 7.3) and fixed overnight at 4 °C in 2.5% glutaraldehyde in PBS. The coverslips were then washed via PBS and dehydrated using EtOH concentrations (25, 50, 75, 95, and 100%) each for 10 min. The plate was left in a desiccator for 24 h to dry. The dried biofilm sample was coated with gold for 2 min using a sputter coater (Quorum, Q150T ES), and examined with an environmental scanning electron microscope (TESCAN-VEGA3, Czech Republic), as described by Kang et al. (2018) [55].

### In silico docking studies

#### 3D structure of proteins for the docking study

The 3D structure (Fig. S1) of several proteins, including enterobactin binding proteins (*FepA* (PDB: 1FEP), *FepB* (PDB: 3TLK)), cell membrane efflux proteins (*AcrB* (PDB: 1T9U) and *TolC* (PDB: 1EK9)), DNA *gyr*ase (PDB: 4KFG), and *MurA* (PDB: 3KQJ), was obtained from the protein data bank (https://www.rcsb.org/).

#### 3D Homology model

The enterobactin-binding proteins (*FepC*, *FepD*, and *FepG*) of *E. coli* OQ866153 were modelled in 3D using a homology-model build server (https://swissmodel.expasy.org/). The modelling process involved aligning the target protein template via the QMEAN scoring function and ProMod3 [56–61].

#### Binding affinity interaction

The docking experiment was carried out using Schrödinger 16.4 with Glide's Extra Precision (XP) program [62]. PubChem Bioassay was used to retrieve enterobactin, Ciprofloxacin, and Fosfomycin for the analysis. ChemBioOffice 14 software was utilized to create Cnj-1 and Cnj-2, as well as Fe^+3^-complexed conjugates. The Maestro 12.8 and LigPrep 2.4 software programs were used to prepare the ligands, with a default grid size of 20 Å for each protein. The energy for all ligands was minimized using the MacroModel of Schrödinger software [63–65].

### Assessment of cytotoxicity and determination of IC50 values in hek293 human embryonic kidney cells

The MTT assay was used to assess the toxic effects of Fe^+3^-Cnj-1, Fe^+3^-Cnj-2, CPF, and Fos compounds on HEK293 human embryonic kidney cells. Different concentrations of each compound, ranging from 10 μg/ml to 100 μg/ml, were applied to the cells for 24 h. The viability of the cells was determined using the MTT assay [66, 67]. The IC50 values, which represent the concentration at which 50% of the cells were affected, were determined using nonlinear regression analysis in GraphPad Prism.

### Supplementary Information

**Supplementary Material 1.**

**Supplementary Material 2.**

## Data Availability

All data generated or analyzed during this study are included in this manuscript and its supplementary information files.

## Abbreviations

AcrB: Acriflavine resistance protein B
ATP: Adenosine triphosphate
CCD: Central composite design
cfu/ml: Colony-forming unit per milliliter
Da: Dalton
DNA: Deoxyribonucleic acid
EPS: Extracellular Polysaccharide
ESEM: Environmental Scanning Electron Microscope
EtBr: Ethidium bromide
EtOH: Ethanol
Fep: Ferric enterobactin
FepABCDG: Enterobactin binding proteins
FTIR: Fourier Transform Infrared Spectroscopy
H-bonds: Hydrogen Bonds
IR: Infrared
LB: Luria-Bertani
MDR: Multidrug resistant
MIC: Minimum Inhibitory Concentration
MHA: Mueller-Hinton agar
MHB: Mueller-Hinton broth
MurA: UDP-N acetylglucosamine 1-carboxyvinyltransferase
NMR: Nuclear Magnetic Resonance
PCR: Polymerase Chain Reaction
PBD: Plackett-Burman design
Pts: Peptide transport system
RND: Resistance nodulation cell division
RSM: Response surface methodology
TolC: Outer membrane protein TolC
UDP: Uracil diphosphate
XP: Extra Precision

## References

[1] Krell T, Matilla MA (2022). Antimicrobial resistance: progress and challenges in antibiotic discovery and anti-infective therapy. Microb Biotechnol.

[2] Alfinete NW, Bolukaoto JY, Heine L, Potgieter N, Barnard TG (2022). Virulence and phylogenetic analysis of enteric pathogenic Escherichia coli isolated from children with diarrhoea in South Africa. Int J Infect Dis.

[3] Rohrbacher C, Zscherp R, Weck SC, Klahn P, Ducho C (2023). Synthesis of an Antimicrobial Enterobactin-Muraymycin Conjugate for Improved Activity Against Gram-Negative Bacteria. Chemistry Eur J..

[4] Akram F, Imtiaz M, Haq IU (2023). Emergent crisis of antibiotic resistance: A silent pandemic threat to 21st century. Microb Pathog.

[5] Salam MA, Al-Amin MY, Salam MT, Pawar JS, Akhter N, Rabaan AA (2023). Antimicrobial Resistance: A Growing Serious Threat for Global Public Health. Healthcare (Switzerland).

[6] Global Action Plan on Antimicrobial Resistance (2015). Microbe Mag..

[7] Centers for Disease Control and Prevention. Antibiotic Resistance Solutions Initiative. Centers for Disease Control and Prevention; 2015. p. 1–2.

[8] Graham-Clarke E, Rushton A, Noblet T, Marriott J (2019). Non-medical prescribing in the United Kingdom National Health Service: a systematic policy review. PLoS One..

[9] Graham-Clarke E, Rushton A, Noblet T, Marriott J. Non-medical prescribing policy in the United Kingdom National Health Service: Systematic review and narrative synthesis. bioRxiv. 2019. 10.1371/journal.pone.0214630 PMC6663007 31356615

[10] Kohl S (2022). Show how you keep antibiotics working during this year’s EAAD. Eur J Hosp Pharm.

[11] Acharya KP, Subramanya SH, Lopes BS (2019). Combatting antimicrobial resistance in Nepal: The need for precision surveillance programmes and multi-sectoral partnership. JAC Antimicrob Resist.

[12] TambićAndrasević A (2004). Antibiotic resistance–bacteria fight back. Acta Med Croatica.

[13] Southwell JW, Black CM, Duhme-Klair AK (2021). Experimental methods for evaluating the bacterial uptake of Trojan horse antibacterials. ChemMedChem.

[14] Huang Y-J, Zhong X-L, Zang Y-P, Yang M-H, Lin J, Chen W-M (2023). 3-Hydroxy-pyridin-4 (1H)-ones as siderophores mediated delivery of isobavachalcone enhances antibacterial activity against pathogenic Pseudomonas aeruginosa. Eur J Med Chem.

[15] Al Shaer D, Al Musaimi O, de la Torre BG, Albericio F (2020). Hydroxamate siderophores: Natural occurrence, chemical synthesis, iron binding affinity and use as Trojan horses against pathogens. Eur J Med Chem.

[16] Kumar A, Yang T, Chakravorty S, Majumdar A, Nairn BL, Six DA (2022). Fluorescent sensors of siderophores produced by bacterial pathogens. J Biol Chem.

[17] Kathayat D, Lokesh D, Ranjit S, Rajashekara G (2021). Avian pathogenic Escherichia coli (APEC): an overview of virulence and pathogenesis factors, zoonotic potential, and control strategies. Pathogens.

[18] Rayner B, Verderosa AD, Ferro V, Blaskovich MAT (2023). Siderophore conjugates to combat antibiotic-resistant bacteria. RSC Med Chem.

[19] Khasheii B, Mahmoodi P, Mohammadzadeh A (2021). Siderophores: Importance in bacterial pathogenesis and applications in medicine and industry. Microbiol Res.

[20] Gupta R, Khan F, Alqahtani FM, Hashem M, Ahmad F. Plant Growth–Promoting Rhizobacteria (PGPR) Assisted Bioremediation of Heavy Metal Toxicity. Appl Biochem Biotechnol. 2023:1–29. 10.1007/s12010-023-04545-3. 10.1007/s12010-023-04545-3 37097400

[21] Molnár Z, Solomon W, Mutum L, Janda T (2023). Understanding the Mechanisms of Fe Deficiency in the Rhizosphere to Promote Plant Resilience. Plants.

[22] Handore A V, Khandelwal SR, Karmakar R, Handore D V. Exploration of bacterial siderophores for sustainable future. In: Climate Change and Microbial Diversity. London: Taylor Francis group, Apple Academic Press; 2022. p. 163–189.

[23] Rosy JC, Ravinarayanan H, Gokila P, Navamuthumani T, Marimuthu SCV, Kunjiappan S (2022). In Silico Screening of Natural Metabolites as Inhibitors of Biosynthesis and Transport of Enterobactin. Biointerface Res Appl Chem.

[24] Delepelaire P (2019). Bacterial ABC transporters of iron containing compounds. Res Microbiol.

[25] Neumann W, Sassone-Corsi M, Raffatellu M, Nolan EM (2018). Esterase-Catalyzed Siderophore Hydrolysis Activates an Enterobactin-Ciprofloxacin Conjugate and Confers Targeted Antibacterial Activity. J Am Chem Soc.

[26] Pham T, Loupias P, Dassonville-Klimpt A, Sonnet P (2019). Drug delivery systems designed to overcome antimicrobial resistance. Med Res Rev.

[27] Bleuel C, Große C, Taudte N, Scherer J, Wesenberg D, Krauß GJ (2005). TolC is involved in enterobactin efflux across the outer membrane of Escheriia coli. J Bacteriol.

[28] Guest RL, Court EA, Waldon JL, Schock KA, Raivio TL (2019). Impaired efflux of the siderophore enterobactin induces envelope stress in Escherichia coli. Front Microbiol.

[29] Shi X, Chen M, Yu Z, Bell JM, Wang H, Forrester I (2019). In situ structure and assembly of the multidrug efflux pump AcrAB-TolC. Nat Commun.

[30] Chowdhury N, Suhani S, Purkaystha A, Begum MK, Raihan T, Alam MDJ (2019). Identification of AcrAB-TolC Efflux Pump Genes and Detection of Mutation in Efflux Repressor AcrR from Omeprazole Responsive Multidrug-Resistant Escherichia coli Isolates Causing Urinary Tract Infections. Microb Insights.

[31] Khazaal MT, El-Hendawy HH, Mabrouk MI, Faraag AHI, Bakkar MR (2022). Antibiotic resistance and siderophores production by clinical Escherichia coli strains. Biotechnologia.

[32] Rodríguez D, González-Bello C (2023). Siderophores: chemical tools for precise antibiotic delivery. Bioorg Med Chem Lett.

[33] Guo Y, Ying Y, Wu Q, Wei B, Chen J, Wang H (2023). β-Cyclopiazonic acid binds iron demonstrating siderophore-like activity and promotes growth in Pseudomonas aeruginosa. J Oceanol Limnol..

[34] Negash KH, Norris JKS, Hodgkinson JT (2019). Siderophore-antibiotic conjugate design: New drugs for bad bugs?. Molecules.

[35] Fardeau S, Dassonville-Klimpt A, Audic N, Sasaki A, Pillon M, Baudrin E (2014). Synthesis and antibacterial activity of catecholate–ciprofloxacin conjugates. Bioorg Med Chem.

[36] Zheng T, Nolan EM (2014). Enterobactin-mediated delivery of ß-lactam antibiotics enhances antibacterial activity against pathogenic escherichia coli. J Am Chem Soc.

[37] Wencewicz TA, Miller MJ (2013). Biscatecholate-monohydroxamate mixed ligand siderophore-carbacephalosporin conjugates are selective sideromycin antibiotics that target Acinetobacter baumannii. J Med Chem.

[38] Ghosh M, Miller PA, Möllmann U, Claypool WD, Schroeder VA, Wolter WR (2017). Targeted antibiotic delivery: selective siderophore conjugation with daptomycin confers potent activity against multidrug resistant Acinetobacter baumannii both in vitro and in vivo. J Med Chem.

[39] Liu R, Miller PA, Vakulenko SB, Stewart NK, Boggess WC, Miller MJ (2018). A synthetic dual drug sideromycin induces Gram-negative bacteria to commit suicide with a Gram-positive antibiotic. J Med Chem.

[40] Schalk IJ (2018). A trojan-horse strategy including a bacterial suicide action for the efficient use of a specific Gram-positive antibiotic on Gram-negative bacteria. J Med Chem.

[41] Lamut A, Cruz CD, Skok Ž, Barančoková M, Zidar N, Zega A (2020). Design, synthesis and biological evaluation of novel DNA gyrase inhibitors and their siderophore mimic conjugates. Bioorg Chem.

[42] Sanderson TJ, Black CM, Southwell JW, Wilde EJ, Pandey A, Herman R (2020). A Salmochelin S4-Inspired Ciprofloxacin Trojan Horse Conjugate. ACS Infect Dis.

[43] Tahmasebi H, Dehbashi S, Nasaj M, Arabestani MR (2022). Molecular epidemiology and collaboration of siderophore-based iron acquisition with surface adhesion in hypervirulent Pseudomonas aeruginosa isolates from wound infections. Sci Rep.

[44] Pybus CA, Felder-Scott C, Obuekwe V, Greenberg DE (2021). Cefiderocol Retains Antibiofilm Activity in Multidrug-Resistant Gram-Negative Pathogens. Antimicrob Agents Chemother..

[45] Flemming H-C, van Hullebusch ED, Neu TR, Nielsen PH, Seviour T, Stoodley P (2023). The biofilm matrix: Multitasking in a shared space. Nat Rev Microbiol.

[46] Díez-Aguilar M, Cantón R (2019). New microbiological aspects of fosfomycin. Rev Esp Quimioter.

[47] Sugathan S, Mandal J (2019). An in vitro experimental study of the effect of fosfomycin in combination with amikacin, ciprofloxacin or meropenem on biofilm formation by multidrug-resistant urinary isolates of Escherichia coli. J Med Microbiol.

[48] Abouwarda AM, Ismail TA, Abu El-Wafa WM, Faraag AHI (2022). Synergistic activity and molecular modelling of fosfomycin combinations with some antibiotics against multidrug resistant Helicobacter pylori. World J Microbiol Biotechnol.

[49] Balouiri M, Sadiki M, Ibnsouda SK (2016). Methods for in vitro evaluating antimicrobial activity: A review. J Pharm Anal.

[50] Hamada MA, Hassan RA, Abdou AM, Elsaba YM, Aloufi AS, Sonbol H (2022). Bio_Fabricated Levan Polymer from Bacillus subtilis MZ292983.1 with Antibacterial, Antibiofilm, and Burn Healing Properties. Applied Sciences (Switzerland).

[51] Foerster S, Unemo M, Hathaway LJ, Low N, Althaus CL (2016). Time-kill curve analysis and pharmacodynamic modelling for in vitro evaluation of antimicrobials against Neisseria gonorrhoeae. BMC Microbiol.

[52] Thorburn CE, Molesworth SJ, Sutherland R, Rittenhouse S (1996). Postantibiotic and post-β-lactamase inhibitor effects of amoxicillin plus clavulanate. Antimicrob Agents Chemother.

[53] Maxwell A, Burton NP, O’Hagan N (2006). High-throughput assays for DNA gyrase and other topoisomerases. Nucleic Acids Res.

[54] Kang J, Jin W, Wang J, Sun Y, Wu X, Liu L (2019). Antibacterial and anti-biofilm activities of peppermint essential oil against Staphylococcus aureus. Lwt.

[55] Kang J, Li Q, Liu L, Jin W, Wang J, Sun Y (2018). The specific effect of gallic acid on Escherichia coli biofilm formation by regulating pgaABCD genes expression. Appl Microbiol Biotechnol.

[56] Waterhouse A, Bertoni M, Bienert S, Studer G, Tauriello G, Gumienny R (2018). SWISS-MODEL: Homology modelling of protein structures and complexes. Nucleic Acids Res.

[57] Guex N, Peitsch MC, Schwede T (2009). Automated comparative protein structure modeling with SWISS-MODEL and Swiss-PdbViewer: A historical perspective. Electrophoresis..

[58] Bienert S, Waterhouse A, De Beer TAP, Tauriello G, Studer G, Bordoli L (2017). The SWISS-MODEL Repository-new features and functionality. Nucleic Acids Res.

[59] Benkert P, Biasini M, Schwede T (2011). Toward the estimation of the absolute quality of individual protein structure models. Bioinformatics.

[60] Bertoni M, Kiefer F, Biasini M, Bordoli L, Schwede T (2017). Modeling protein quaternary structure of homo- and hetero-oligomers beyond binary interactions by homology. Sci Rep.

[61] Remmert M, Biegert A, Hauser A, Söding J (2012). HHblits: Lightning-fast iterative protein sequence searching by HMM-HMM alignment. Nat Methods.

[62] Friesner RA, Murphy RB, Repasky MP, Frye LL, Greenwood JR, Halgren TA (2006). Extra precision glide: Docking and scoring incorporating a model of hydrophobic enclosure for protein-ligand complexes. J Med Chem.

[63] Jorgensen WL, Maxwell DS, Tirado-Rives J (1996). Development and testing of the OPLS all-atom force field on conformational energetics and properties of organic liquids. J Am Chem Soc.

[64] Kaminski GA, Friesner RA, Tirado-Rives J, Jorgensen WL (2001). Evaluation and reparametrization of the OPLS-AA force field for proteins via comparison with accurate quantum chemical calculations on peptides. J Phys Chem B.

[65] Schrodinger LLC. MacroModel, Version 10.2. New York (NY), USA. 2013.

[66] Buch K, Peters T, Nawroth T, Sänger M, Schmidberger H, Langguth P (2012). Determination of cell survival after irradiation via clonogenic assay versus multiple MTT Assay - A comparative study. Radiat Oncol.

[67] Moradi M (2018). Determining optimal cell density and culture medium volume simultaneously in MTT cell proliferation assay for adherent cancer cell Lines. HELIX..

