# Supplementary material for: In vitro and in silico studies of enterobactin-inspired Ciprofloxacin and Fosfomycin first generation conjugates on the antibiotic resistant E. coli OQ866153
Source: BMC Microbiol. 2024 Mar 22;24:95. doi: 10.1186/s12866-024-03248-x (PMC10958948; doi:10.1186/s12866-024-03248-x)

**Supplementary data:**

**Fig. S1.** The 3D structure of enterobactin binding proteins (*FepA* (PDB: 1FEP)*, FepB* (PDB: 3TLK)), cell membrane efflux proteins (*AcrB* (PDB: 1T9U) and *TolC* (PDB: 1EK9)), DNA *gyr*ase (PDB: 4KFG), and *MurA* (PDB: 3KQJ) retrieved from protein data bank (<https://www.rcsb.org/>).

**Fig. S2. FTIR spectrum at a 400–4000 cm^-1^ wavelength range with a resolution of 4 cm^-1^.** **(A)**, ciprofloxacin and **(B)**, Fosfomycin.

**Fig. S3.** The 3D homology model of *E. coli* *FepC, FepD,* and *FepG* proteins.


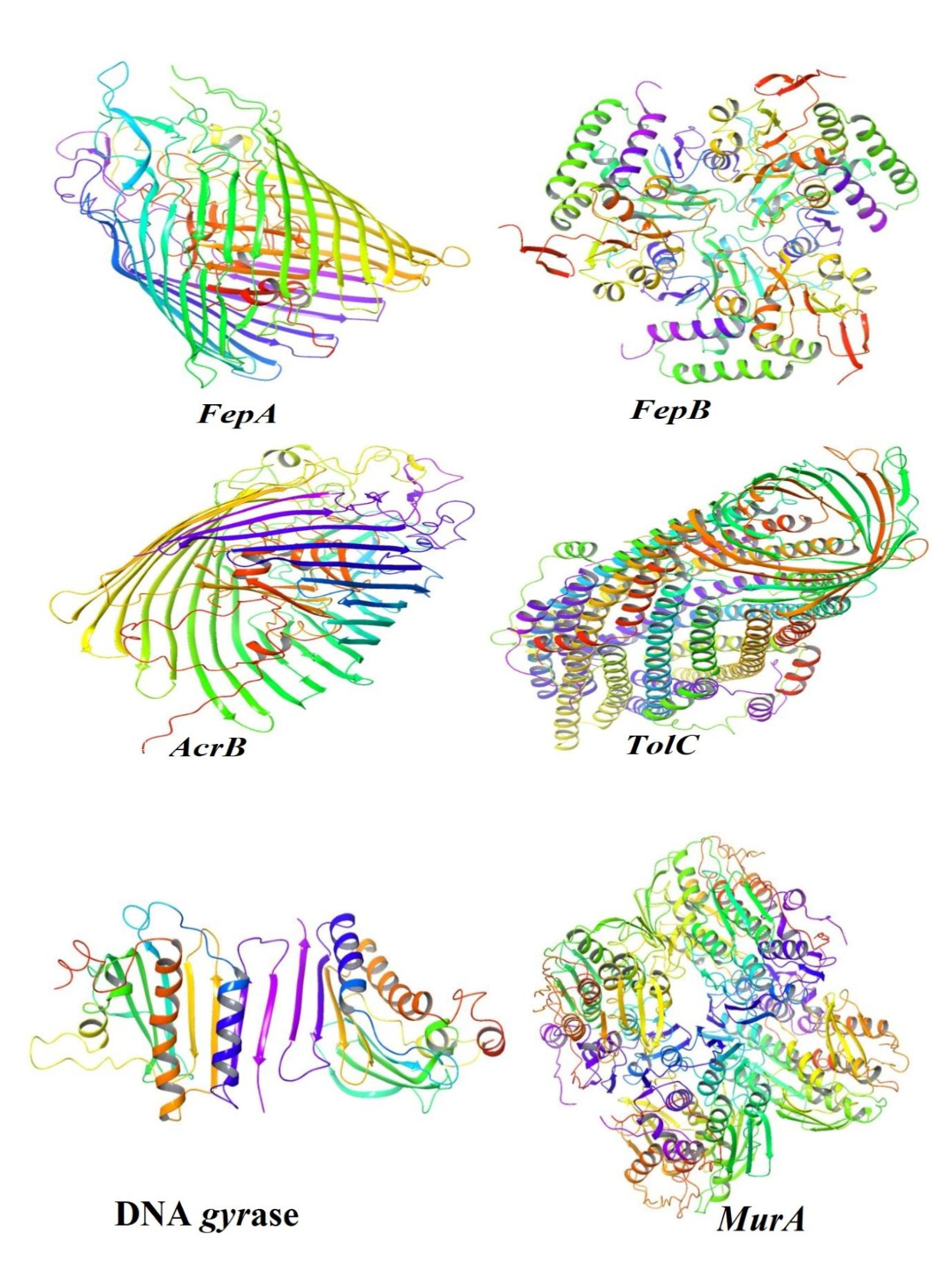


**Fig. S1.** The 3D structure of enterobactin binding proteins (*FepA* (PDB: 1FEP)*, FepB* (PDB: 3TLK)), cell membrane efflux proteins (*AcrB* (PDB: 1T9U) and *TolC* (PDB: 1EK9), DNA *gyr*ase (PDB: 4KFG), and *MurA* (PDB: 3KQJ) retrieved from protein data bank (<https://www.rcsb.org/>).


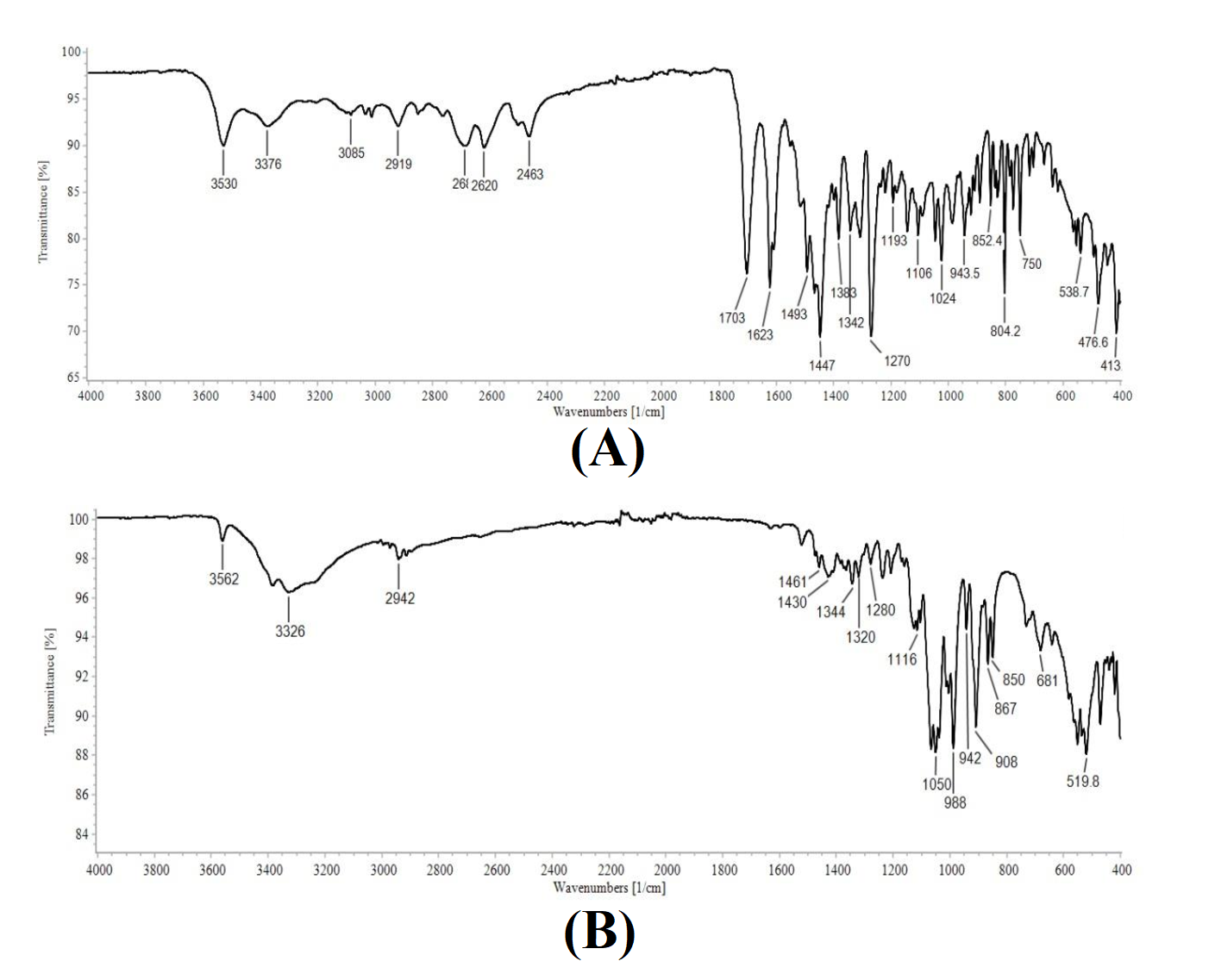
 **Fig. S2. FTIR spectrum at a 400–4000 cm^-1^ wavelength range with a resolution of 4 cm^-1^.** **(A)**, ciprofloxacin and **(B)**, Fosfomycin sodium salt.


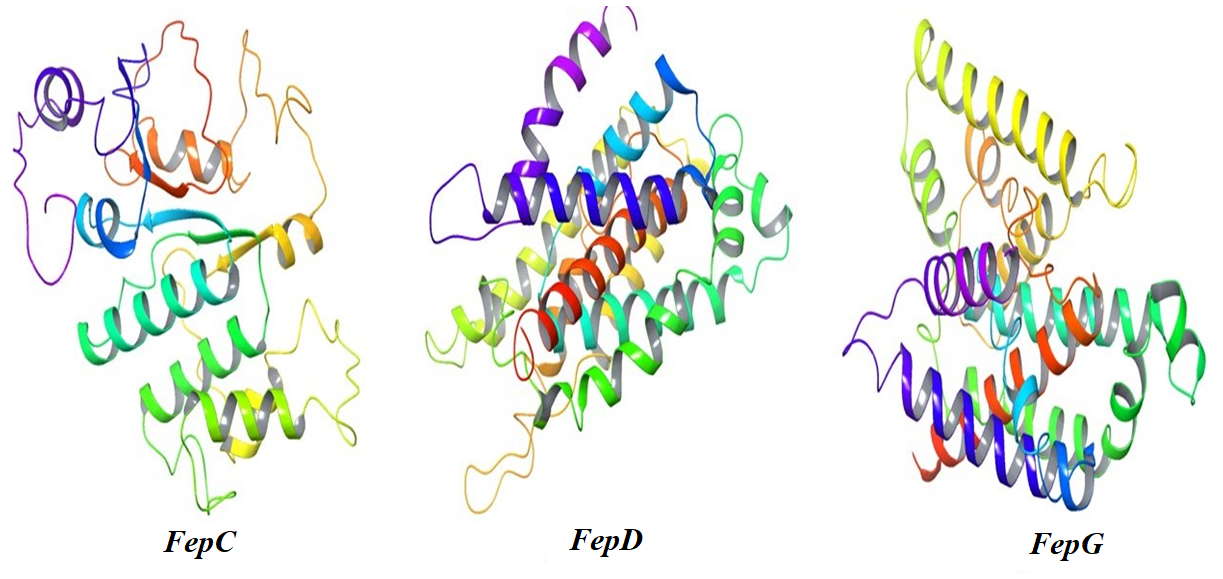
**Fig. S3.** The 3D homology model of *E. coli* *FepC, FepD,* and *FepG* proteins.

Figure 1 (A)
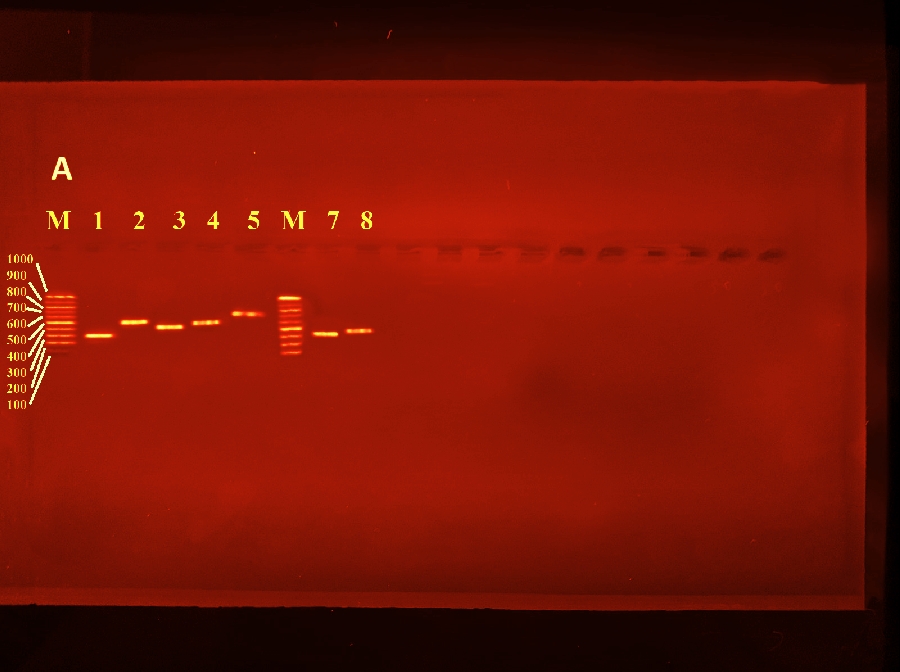


Figure 1(B)


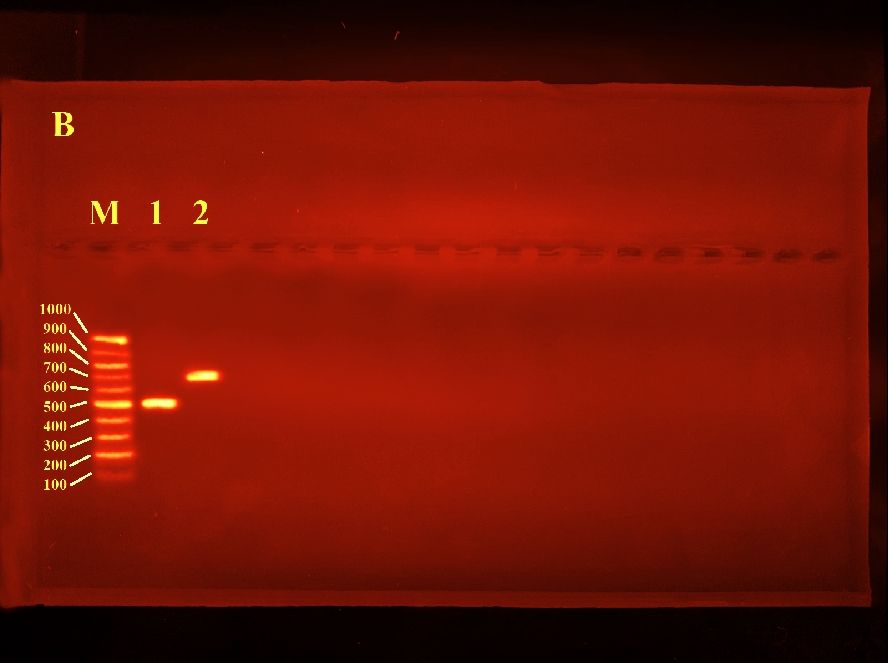


Figure 4


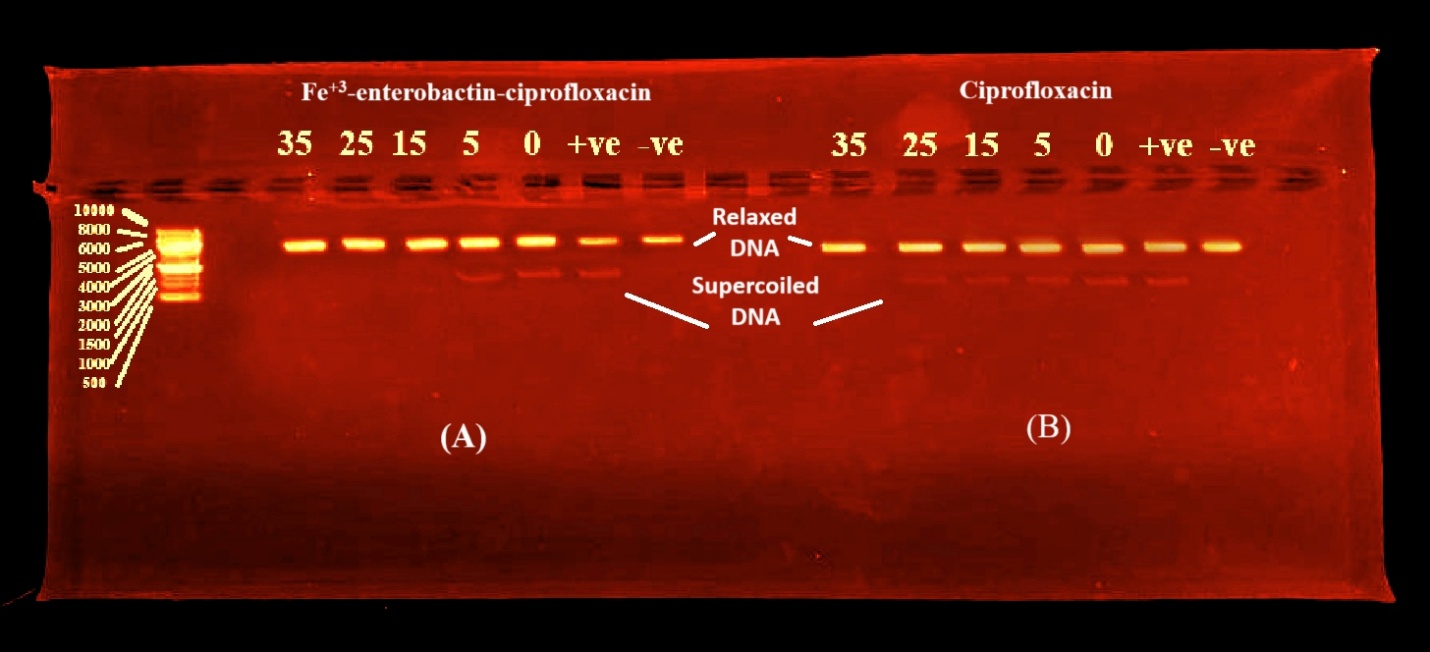

Supplement: Supplementary file 1 — Supplementary Material 1. [file 12866_2024_3248_MOESM1_ESM.docx]
