# Supplementary material for: In vitro and in silico studies of enterobactin-inspired Ciprofloxacin and Fosfomycin first generation conjugates on the antibiotic resistant E. coli OQ866153
Source: BMC Microbiol. 2024 Mar 22;24:95. doi: 10.1186/s12866-024-03248-x (PMC10958948; doi:10.1186/s12866-024-03248-x)
Supplement: Supplementary file 2 — Supplementary Material 2. [file 12866_2024_3248_MOESM2_ESM.docx]

**Table S1.** One-way ANOVA followed by Dunnett’s post hoc test time-kill kinetics data analysis of untreated (control), Ciprofloxacin (CPF), and Fe^+3^- Enterobactin -Ciprofloxacin (Fe^+3^-Cnj-1) against *E. coli* OQ866153.

| Table Analyzed | Time kill kinetics |  |  |  |  |
| --- | --- | --- | --- | --- | --- |
| ANOVA summary |  |  |  |  |  |
| F | 19.23 |  |  |  |  |
| P value | **<0.0001** | **(Significant)** |  |  |  |
| P value summary | **** |  |  |  |  |
| Significant diff. among means (P < 0.05)? | Yes |  |  |  |  |
| R squared | 0.7194 |  |  |  |  |
| Brown-Forsythe test |  |  |  |  |  |
| F (DFn, DFd) | 0.1271 (2, 15) |  |  |  |  |
| P value | 0.8816 |  |  |  |  |
| P value summary | ns |  |  |  |  |
| Are SDs significantly different (P < 0.05)? | No |  |  |  |  |
| Bartlett's test |  |  |  |  |  |
| Bartlett's statistic (corrected) | 0.9719 |  |  |  |  |
| P value | 0.6151 |  |  |  |  |
| P value summary | ns |  |  |  |  |
| Are SDs significantly different (P < 0.05)? | No |  |  |  |  |
| ANOVA table | SS | DF | MS | F (DFn, DFd) | P value |
| Treatment (between columns) | 42.03 | 2 | 21.01 | F (2, 15) = 19.23 | **P<0.0001** |
| Residual (within columns) | 16.39 | 15 | 1.093 |  |  |
| Total | 58.42 | 17 |  |  |  |

**Table S2.** One-way ANOVA followed by Dunnett’s post hoc test time-kill kinetics data analysis of untreated (control), Fosfomycin (FOS), and Fe^+3^**-**Enterobactin-Fosfomycin **(**Fe^+3^-Cnj-2**)** against *E. coli* OQ866153**.**

| Table Analyzed | Time kill kinetics |  |  |  |  |
| --- | --- | --- | --- | --- | --- |
| ANOVA summary |  |  |  |  |  |
| F | 16.85 |  |  |  |  |
| P value | **0.0001** | **(Significant)** |  |  |  |
| P value summary | *** |  |  |  |  |
| Significant diff. among means (P < 0.05)? | Yes |  |  |  |  |
| R squared | 0.6920 |  |  |  |  |
|  |  |  |  |  |  |
| Brown-Forsythe test |  |  |  |  |  |
| F (DFn, DFd) | 6.535 (2, 15) |  |  |  |  |
| P value | 0.0091 |  |  |  |  |
| P value summary | ** |  |  |  |  |
| Are SDs significantly different (P < 0.05)? | Yes |  |  |  |  |
|  |  |  |  |  |  |
| Bartlett's test |  |  |  |  |  |
| Bartlett's statistic (corrected) | 7.218 |  |  |  |  |
| P value | 0.0271 |  |  |  |  |
| P value summary | * |  |  |  |  |
| Are SDs significantly different (P < 0.05)? | Yes |  |  |  |  |
|  |  |  |  |  |  |
| ANOVA table | SS | DF | MS | F (DFn, DFd) | P value |
| Treatment (between columns) | 61.19 | 2 | 30.59 | F (2, 15) = 16.85 | P=0.0001 |
| Residual (within columns) | 27.23 | 15 | 1.816 |  |  |
| Total | 88.42 | 17 |  |  |  |
|  |  |  |  |  |  |
